# HiST: Histological Images Reconstruct Tumor Spatial Transcriptomics via MultiScale Fusion Deep Learning

**DOI:** 10.1002/advs.202514351

**Published:** 2026-01-05

**Authors:** Wei Li, Dong Zhang, Eryu Peng, Shijun Shen, Hamid Alinejad‐Rokny, Yao Liu, Junke Zheng, Cizhong Jiang, Youqiong Ye

**Affiliations:** ^1^ Shanghai Tenth People's Hospital Shanghai Key Laboratory of Signaling and Disease Research School of Life Sciences and Technology Tongji University Shanghai China; ^2^ Shanghai Institute of Immunology Department of Immunology and Microbiology Shanghai Jiao Tong University School of Medicine Shanghai China; ^3^ Department of Hepatobiliary Surgery State Key Laboratory of Immune Response and Immunotherapy Centre For Leading Medicine and Advanced Technologies of IHM The First Affiliated Hospital of USTC Division of Life Sciences and Medicine University of Science and Technology of China Hefei Anhui China; ^4^ Institute for Translational Medicine On Cell Fate and Disease Shanghai Ninth People's Hospital Key Laboratory of Cell Differentiation and Apoptosis of National Ministry of Education Department of Pathophysiology Shanghai Jiao Tong University School of Medicine Shanghai China; ^5^ UNSW BioMedical Machine Learning Lab (BML) School of Biomedical Engineering UNSW Sydney Sydney NSW Australia

**Keywords:** HiST, histological image, prognosis and immunotherapy efficacy prediction, spatial transcriptomics, tumor spot identification

## Abstract

Spatial transcriptomics (ST) provides valuable insights into the tumor microenvironment by integrating molecular features with spatial context; however, its clinical utility is limited by high costs. To address this, we develop a multi‐scale convolutional deep learning framework, HiST, which utilizes ST to learn the relationship between spatially resolved gene expression profiles (GEPs) and histological morphology. HiST accurately predicts tumor regions across multiple cancer types (e.g., breast cancer; area under the curve: 0.96), demonstrating high concordance with pathologist annotations. Moreover, HiST reconstructs spatially resolved GEPs from histological images with an average Pearson correlation coefficient of 0.74 across five cancer types, outperforming existing models by about two‐fold. These high‐fidelity spatial GEPs enable tumor heterogeneity assessment from histological images, including identification of tumor subtypes with distinct DNA copy number variations. We demonstrate the clinical utility of the predicted GEPs, which robustly stratify patient prognosis across five cancer types from The Cancer Genome Atlas (e.g., breast cancer; concordance index: 0.78). The predicted profiles further facilitate immunotherapy response prediction and enrichment analyses of relevant biological pathways and markers. Collectively, HiST achieves state‐of‐the‐art performance in spatial GEP reconstruction, providing a reliable molecular representation that enhances downstream tasks such as tumor profiling and clinical analyses.

## Graphical abstract

HiST, a multiscale deep learning framework, reconstructs spatially resolved gene expression profiles directly from histological images. It accurately identifies tumor regions, captures intratumoral heterogeneity, and predicts patient prognosis and immunotherapy response. By bridging histopathology and spatial transcriptomics, HiST offers a cost‐effective approach to advance precision oncology and spatial cancer profiling.

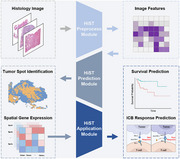

## 1 Introduction

In clinical practice, pathologists diagnose cancer by analyzing tissue structure and cellular abnormalities in histological images, such as Hematoxylin and Eosin (HE)‐stained sections [1, 2]. However, the complexity and diversity of stained images, coupled with differences in pathologist experience and subjectivity, may lead to diagnostic inconsistencies [3, 4, 5, 6]. Moreover, the tumor microenvironment is heterogeneous and involves molecular regulation at various levels, making it challenging for histological images alone to provide a comprehensive assessment of disease states [7, 8]. The integration of spatial transcriptomics (ST) with HE‐stained images has enabled the quantification of gene expression at spatial coordinates across tissues, offering key molecular insights into disease pathology [9, 10, 11, 12, 13, 14, 15, 16]. However, the high cost and long processing time associated with ST currently limit its feasibility as a routine diagnostic tool [17, 18, 19, 20]. Advances in artificial intelligence (AI) have allowed the use of paired ST and HE‐stained images as training datasets to predict spatial gene expression profiles (GEPs) directly from HE‐stained images [21, 22, 23].

To date, several methods have been developed to predict spatial GEPs using only HE‐stained images. ST‐Net [24], based on a fine‐tuned DenseNet‐121 model, was trained on ST data from 68 breast tissue sections to predict the expression of 250 genes. Due to the large size of whole‐slide images (WSIs), splitting slides into smaller patches, with predictions relying on integrating patch information, was required. To address this, Pang et al. introduced HisToGene [25], a Vision Transformer (ViT)‐based model using self‐attention to capture relationships between spatial spots. Zeng et al. proposed Hist2ST [26], which combined convolutional layers, ViT, and a graph neural network (GNN), later refined in THItoGene [27] by replacing GNN with a graph attention network. While ViT‐based models improve global contextual understanding via position encoding, absolute position embeddings do not effectively represent the relative spatial relationships of patches. Furthermore, the self‐attention mechanism in Transformer models requires extensive parameters, further increasing susceptibility to overfitting due to the limited size of ST datasets. A recent benchmarking study compared eleven methods for predicting spatial GEPs from histological images [28]. Although EGNv2 [29] demonstrated the best overall performance, its average Pearson correlation coefficient (PCC) between ground truth and predicted gene expression was only 0.28. This study did not include IGI‐DL [8], a more recent tool that achieved a higher average PCC of 0.34. IGI‐DL integrates patch‐derived nuclei graphs and convolutional features for spatial gene expression prediction but fails to account for the global spatial context between patches across the WSI. Furthermore, inaccurate gene expression prediction indicates a failure to capture essential molecular features, limiting the reliability of any downstream analyses.

To overcome the limitations of existing tools for spatial GEP prediction from histological images, we developed Histological Images reconstructs tumor Spatial Transcriptomics via multi‐scale fusion deep learning (HiST), a convolution‐based framework comprising three modules: preprocessing, prediction, and application. To capture the global histological context while preserving intra‐patch features, the preprocessing module extracts features to generate a feature map that represents WSI information while maintaining the relative positions of original patches. The prediction module uses these spatial features to accurately identify tumor regions and reconstruct spatial transcriptomic profiles. Benchmarking the performance of the HiST against publicly available models demonstrated that it not only precisely segments tumor regions within histological images but also outperforms existing models in predicting spatial GEPs across multiple cancer types. Moreover, HiST distinguishes tumor heterogeneity within the same slide. The application module provides proof‐of‐concept analyses showing that HiST's predicted spatial GEPs retain clinically relevant signals, enabling prognosis and immunotherapy response prediction.

## 2 Results

### 2.1 HiST Model Structure

**FIGURE 1:**
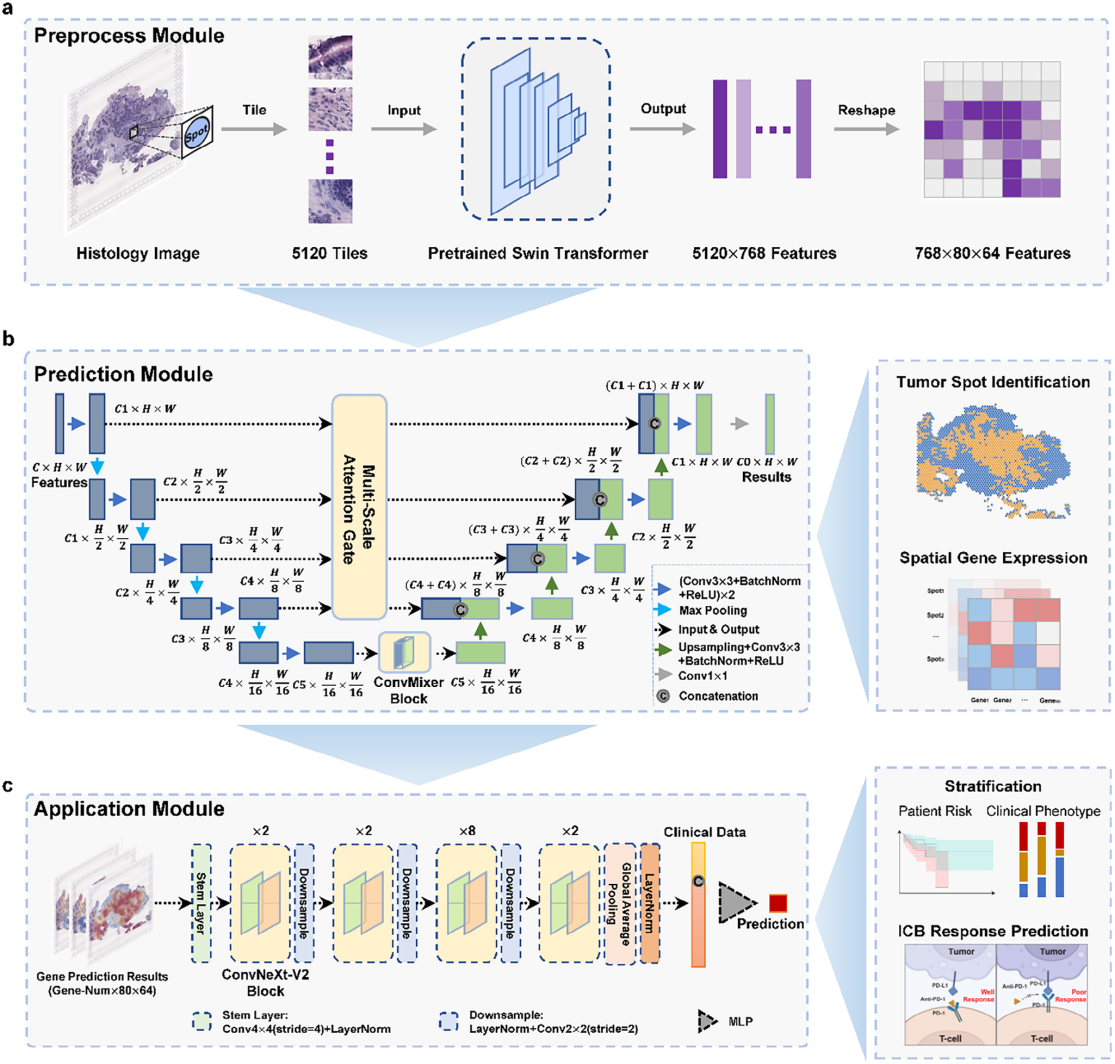
Schematic illustration of the HiST framework. a) Workflow of preprocessing histology images to feature maps. b) U‐Net based network structure of prediction module (left). Outputs of prediction module (right). C, channel; H, height; W, width. c) ConvNeXt‐V2 based network structure of the application module (left). Application of HiST (right). Conv, convolutional kernel; LayerNorm, layer normalization; MLP, multilayer perceptron. Created with BioRender.com.

HiST was developed to reconstruct tumor ST from HE‐stained histological images using a multi‐scale fusion deep learning framework consisting of three modules: (i) preprocessing, (ii) prediction, and (iii) application. The preprocessing module extracts histological information and spatial context from the original WSI while minimizing the high GPU memory requirements of high‐resolution WSIs (Figure 1a). Briefly, HE‐stained histological images were segmented from the ST data based on spot locations and cropped into patches matching the spot size. A Swin Transformer‐based model pre‐trained on large‐scale pathological image datasets was then used to extract features from each patch [30]. Feature extraction effectively converts each patch into a low‐dimensional feature vector, capturing key morphological information. To integrate spatial background and align image features with spot locations, the feature vector was reshaped into a feature map scaled to the original image, preserving histological information while reducing computational complexity.

The prediction module is based on an improved U‐Net [31] framework designed for two primary tasks: tumor spot prediction and tumor ST reconstruction (Figure 1b; Figure S1). Multi‐scale attention gate skip connections were introduced to address U‐Net's limitation in capturing global context. The encoder consists of five convolutional levels, each containing two convolutional blocks composed of a convolutional layer, batch normalization, a ReLU activation function, and a downsampling layer. The decoder is symmetric to the encoder and is connected via a multi‐scale attention gate (Figure S1) to improve feature filtering. Features from the last convolutional level are further processed through a ConvMixer module (Figure S1), which applies repeated depth‐wise and pointwise convolutions to improve feature extraction. This overall design improves tumor spot segmentation through multi‐scale feature extraction and attention mechanisms. For predicting spatial gene expression, the segmentation model architecture was adapted into a regression model to output a spot‐resolved spatial expression matrix, where each channel corresponds to the spatial GEP of a specific gene.

The application module is built on ConvNeXt‐V2 [32] (Figure 1c; Figure S1) and uses ST profiles predicted by the model as molecular features of HE‐stained histological images. The model architecture consists of a stem block for initial data processing, followed by four stages of ConvNeXt‐V2 blocks with downsampling layers for progressively extracting high‐level features. A global average pooling layer combined with layer normalization aggregates spatial information and stabilizes training, followed by a linear layer for feature representation output. For survival prediction, features extracted from convolutional blocks were concatenated with one‐hot encoded clinical data and input into a multilayer perceptron (MLP) for prognosis prediction. The model was trained for disease prognosis and immune response prediction using a supervised learning strategy.

### 2.2 HiST Accurately Predicts Tumor Spots Among Multiple Cancers

**FIGURE 2:**
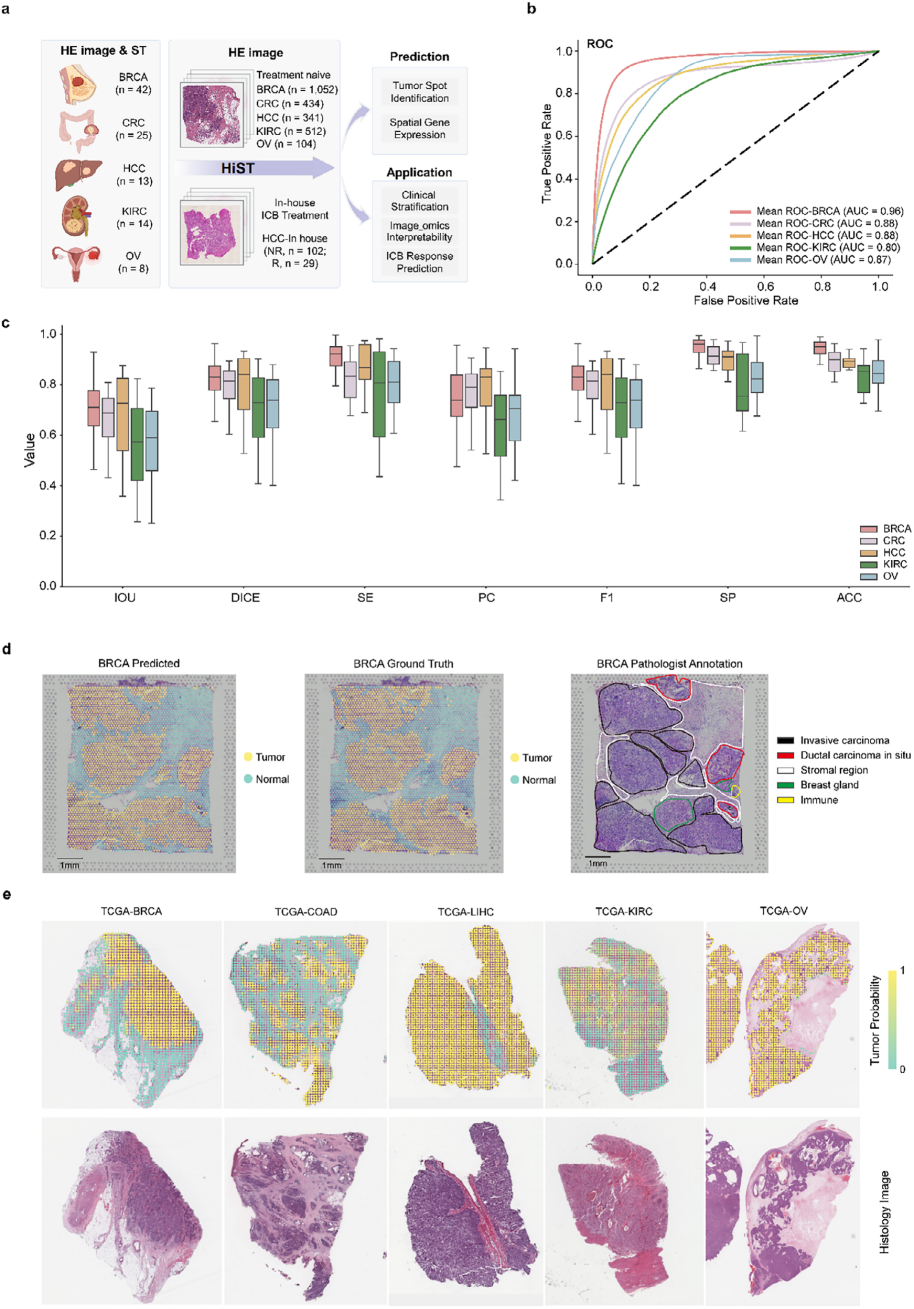
Predicted tumor region results of HiST in the training datasets and validation datasets. a) Overview of primary datasets and sample sizes used in this study (created with BioRender.com). NR, non‐responder; R, responder; BRCA, breast cancer; CRC, colorectal cancer; HCC. hepatocellular carcinoma; KIRC, kidney renal clear cell carcinoma; OV, ovarian cancer. b) ROC curves of the predicted tumor regions of the five types of cancer on the training datasets. c) The IOU, DICE, SE, PC, F1, SP and ACC of the five cancer types. IOU, intersection over union; DICE, dice similarity coefficient; SE, standard error; PC, precision; SP, specificity; ACC, accuracy. d) For the same BRCA sample, HiST predicted tumor regions are shown on the left, Cottrazm annotated tumor regions are shown in the middle, and tissue annotations by pathologists are shown on the right. e) HiST‐predicted tumor probability on TCGA pathology images.

HiST was trained using 102 ST slides from five cancer types, including breast cancer (BRCA, *n* = 42), colorectal cancer (CRC, *n* = 25; public data, *n* = 2; in‐house data, *n* = 23), hepatocellular carcinoma (HCC, *n* = 13), kidney renal clear cell carcinoma (KIRC, *n* = 14), and ovarian cancer (OV, *n* = 8) (Figure 2a; Table S1). Tumor‐containing spots were identified as the ground truth for tumor regions using Cottrazm [33], a previously developed tool. The Cottrazm‐BoundaryDefine output was used to calculate the loss value in HiST's model training process. HiST's performance in predicting tumor regions was evaluated using multiple metrics. Receiver operating characteristic (ROC) curves demonstrated HiST predictive accuracy across five cancer types, with area under the curve (AUC) values ranging from 0.80 in KIRC to 0.96 in BRCA (Figure 2b; Figure S2). Other evaluation metrics, including intersection over union (IOU), Dice similarity coefficient, standard error (SE), precision (PC), F1 score, specificity (SP), and accuracy (ACC), further validated the model's robustness. The average median ACC across the five cancer types was greater than 0.80, with the average median value of F1 and other indicators above 0.60 (Figure 2c), indicating high sensitivity and precision in tumor region identification. Pathologist‐annotated HE‐stained histological images demonstrated that HiST accurately predicted tumor regions identified by Cottrazm, as well as other tumor regions not marked by Cottrazm, suggesting its ability to capture subtle tumor features (Figure 2d; Figure S3). Furthermore, HE‐stained images from The Cancer Genome Atlas (TCGA) served as independent test datasets, where HiST‐predicted tumor regions exhibited high concordance with cancerous regions in pathological tissues, as validated by pathologists (Figure 2e). These findings indicate that HiST performs well on both training and test datasets, demonstrating strong generalization ability.

### 2.3 Benchmarking HiST and Existing Methods for Reconstructing Tumor Spatial Transcriptomics

To evaluate HiST's performance in predicting gene expression, we compared it against six spatial GEPs prediction models: ST‐Net, HisToGene, Hist2ST, THItoGene, IGI‐DL and EGNv2. To ensure spatial relevance in prediction targets, spatial autocorrelation was calculated across all samples, selecting spatially variable genes for evaluation. This approach ensured that the selected genes exhibited meaningful spatial patterns. Across all benchmark models, the target genes remained consistent when performing on the same cancer type dataset, enabling comparability and reproducibility of results. The number of target genes used for each dataset is listed in Table S2.

**FIGURE 3:**
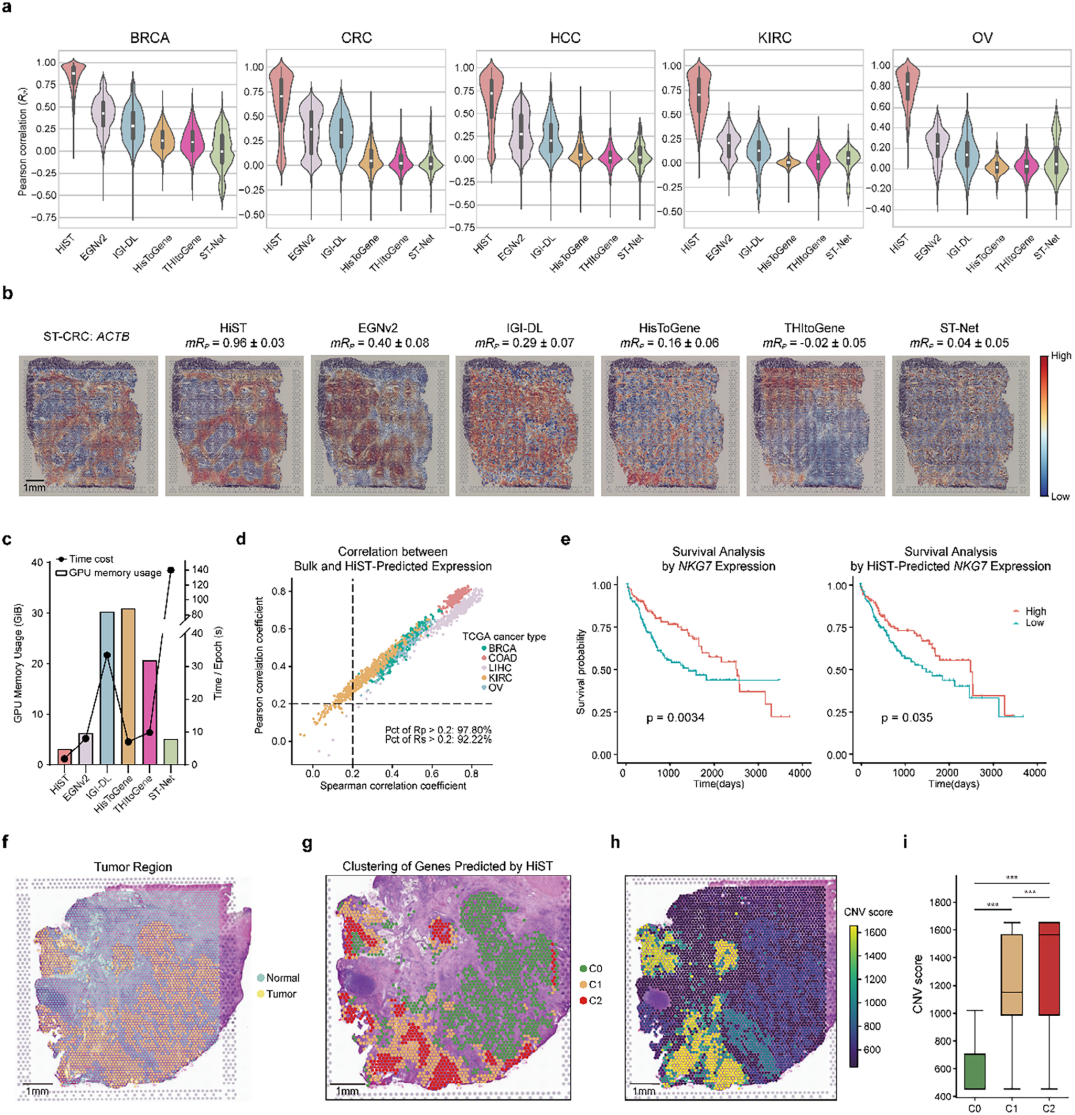
The performance of HiST on reconstructing spatial GEPs. a) Leave‐one‐out validation Pearson correlation performance of 6 models for spatial gene expression prediction on 5 ST datasets. b) Visualization of ground truth and predicted *ACTB* spatial gene expression on CRC ST sample (scale bar, 1 mm). Color bar indicates the gene expression level. *mR_p_* ± 95% CI denotes the mean Pearson correlation coefficient across cross‐validation folds together with its 95% confidence interval computed using the Student's *t*‐test. c) The time cost and GPU memory usage of different models. d) Spearman and Pearson correlation between RNA‐seq and mean of HiST‐predicted GEPs. e) Kaplan‐Meier curve comparing overall survival by *NKG7* from RNA‐seq Expression (left). Kaplan‐Meier curve and by means of *NKG7* from HiST‐predicted spatial expression in TCGA LIHC cohort (right). f) Spatial plot showing CRC ST sample tumor distribution (scale bar, 1 mm). g) Clustering of genes predicted by HiST in tumor region (scale bar, 1 mm). h) Spatial plot showing CNV score of CRC ST sample (scale bar, 1 mm). i) CNV score comparison between HiST‐predicted 3 clusters. Log rank test was used in e, two‐sided Wilcoxon test was used in i.

To assess model generalization, leave‐one‐out cross‐validation (LOOCV) was performed across all samples. In each cancer type, the Pearson correlation coefficient (*R_p_*) between the predicted and ground truth spatial GEPs was compared across models (Figure 3a; Figure S4; Table S2). HiST consistently outperformed existing models, achieving mean *R_p_* values of 0.83 ± 0.04 in BRCA, 0.62 ± 0.04 in CRC, 0.63 ± 0.06 in HCC, 0.68 ± 0.09 in KIRC, and 0.79 ± 0.07 in OV, with the 95% confidence interval calculated using Student's t‐test. On the other hand, the second‐ranked model, EGNv2, achieved mean *R_p_* values of only 0.41 ± 0.05, 0.34 ± 0.05, 0.29 ± 0.08, 0.19 ± 0.07 and 0.22 ± 0.11 for the corresponding cancer types. The average *R_p_* of the HiST's gene prediction model showed a significant improvement, outperforming the spatial GEP prediction models published to date by about two‐fold. Taking the dysregulated gene *ACTB* [34] in CRC as an example, the mean *R_p_* between HiST‐predicted *ACTB* spatial expression and ground truth from the ST dataset reached 0.96 ± 0.03, whereas EGNv2, IGI‐DL, HisToGene, THItoGene, and ST‐Net achieved only 0.40 ± 0.08, 0.29 ± 0.07, 0.16 ± 0.06, ‐0.02 ± 0.05 and 0.04 ± 0.05, respectively (Figure 3b). Similarly, the mean correlation for *FTL* gene expression predicted by HiST reached 0.97 ± 0.01 and 0.97 ± 0.02 in BRCA and HCC, respectively (Figure S5a). Comparable performance was observed in KIRC and OV, with *UBC* (mean *R_p_* = 0.93 ± 0.03) in KIRC and *FTH1* (mean *R_p_* = 0.98 ± 0.01) in OV, showing results similar to ground truth. Given that *ACTB* [34], *FTL* [35, 36], *UBC* [37], and *FTH1* [38] have been associated with poor prognosis or disease progression, these results suggest that HiST's ability to predict gene expression patterns from HE images has potential clinical diagnostic applications.

Due to the substantial GPU memory demands imposed by Hist2ST's Transformer‐based architecture, it could not be trained on the 10× Visium dataset, limiting its applicability to high‐resolution ST data. Training speed and GPU memory usage of HiST alongside five other spatial gene prediction models were evaluated under identical experimental conditions using the HCC dataset (Figure 3c). Using the HiST's preprocessing module, the feature dimensions of histology images were effectively compressed through feature extraction while preserving spatial relationships. Compared to ViT and GNN‐based models, the convolutional architecture of HiST offered improved computational efficiency, achieving high predictive accuracy with fewer model parameters.

In addition, prediction performance was evaluated on HER2 positive breast cancer and cutaneous squamous‐cell carcinoma (cSCC) datasets derived from legacy ST technologies (Figure S6a,b). The mean *R_p_* between HiST‐predicted and ground truth spatial GEPs reached 0.45 ± 0.04 and 0.41 ± 0.08 in HER2 and cSCC datasets, respectively, outperforming the second‐best method by 0.28 and 0.17, respectively. Visualization of spatial gene expression predictions further confirmed HiST's higher predictive ability (Figure S5b), highlighting its robustness in predicting spatial gene expression across multiple cancer types.

We further assessed generalizability across cohorts, sequencing platforms and unseen patients. Models trained using LOOCV on the 10X Visium‐based ST from BRCA cohort were directly applied to the HER2 samples to predict spatial GEPs. HiST achieves a mean *R_p_* of 0.52 ± 0.04 (Figure S6c), indicating strong robustness to technical and biological variability.

Beyond Visium‐based HE‐to‐spatial‐transcriptomic prediction frameworks, we further compared HiST with methods developed for bulk‐level or higher‐resolution transcriptomic prediction (Figure S7a), including iStar [39], SEQUOIA [40], and hist2RNA [41]. At the spot level, we benchmarked HiST against iStar and an adapted SEQUOIA model with its aggregation module removed to enable direct patch‐to‐spot inference. HiST consistently achieved the highest per‐gene Pearson correlation across all cancer types (Figure S7b), outperforming SEQUOIA and substantially exceeding iStar, whose performance demonstrated limited generalization across slides. At the bulk level, we additionally evaluated hist2RNA and SEQUOIA using a pseudo‐bulk strategy. HiST's aggregation of fine‐grained spot‐level predictions produced more accurate whole‐slide expression profiles than both WSI‐to‐bulk models, including SEQUOIA with pretrained weights (Figure S7c). Together, these results highlight that modeling spatial heterogeneity at spot resolution confers clear advantages, even when the final task is bulk‐level expression prediction.

To further validate HiST's clinical application and performance, the correlation between HiST‐predicted spatial GEPs from TCGA patient histology WSIs and bulk RNA‐seq expression was analyzed. To align with RNA‐seq data, predicted expression values were averaged across the entire slide to generate HiST‐derived pseudo‐RNA‐seq expression for each patient. Expression profiles corresponding to the cancer types and genes predicted by HiST were extracted from the TCGA database. After filtering, 1,050, 306, 335, 509, and 68 samples were retained for BRCA, colon adenocarcinoma (COAD), liver hepatocellular carcinoma (LIHC), KIRC, and OV, respectively, with 426, 263, 408, 172, and 134 genes included for subsequent Pearson and Spearman correlation analyses. Across all samples, 97.80% of patients had a Pearson correlation coefficient (*R_p_*) > 0.2, while 92.22% of patients had a Spearman correlation coefficient (*R_s_*) > 0.2 (Figure 3d). Among the five cancer types, COAD and LIHC showed the highest prediction performance, with an average *R_p_* of 0.76 and 0.67, respectively. *NKG7* is a well‐established marker of NK cell activity and has been previously associated with tumor prognosis [42]. Using TCGA‐LIHC clinical data and HiST‐predicted pseudo‐RNA‐seq expression, survival analysis demonstrated that higher *NKG7* expression in LIHC patients was associated with better prognosis, consistent with survival analysis results based on RNA‐seq data (Figure 3e).

Targeted ablation experiments were performed to evaluate the contribution of HiST's key architectural components. To examine the effect of spatial arrangement, we performed an ablation study using five 10X Visium datasets, where the spatial positions of patch features were randomly shuffled before aggregation. The results showed a significant decline in HiST's predictive performance when spatial dependencies were disrupted (Figure S8a), emphasizing the importance of maintaining the relative spatial arrangement of tissue patches. In addition to spatial topology preservation, HiST incorporates a multi‐scale attention gate (MSAG) to model complex spatial dependencies at different scales. An additional ablation study (Figure S8a) was carried out to assess the contribution of this component. Replacing the MSAG with a simple skip connection resulted in a noticeable decrease in performance, and removing all skip connections caused a considerable degradation. These findings indicate that the MSAG's adaptive gating mechanism enhances the model's ability to capture multi‐scale spatial contexts and that skip connections are essential for maintaining gradient flow and ensuring accurate morphology‐gene expression associations.

To further elucidate these interactions, we generated Grad‐CAM‐based attribution maps to visualize the histological regions most influential in gene expression predictions. For instance, in the prediction of *ACTB* expression in a colorectal cancer (CRC) sample (Figure 3a), we present attribution panels created from the last layer of each block in HiST using Grad‐CAM (Figure S8b). The attribution maps from Grad‐CAM provide insight into the contribution of different components of HiST to gene expression predictions. In the encoder (upper panel of Figure S8b), shallow layers (Conv1–Conv5) focus on tissue boundaries and local textures, while deeper layers capture tissue‐specific regions, forming larger, more uniform areas of high activation. In the skip connection (middle panel of Figure S8b), the MSAG1‐MSAG4 modules help transfer local morphological information from shallow to deep layers, showing continuous high activation in intermediate regions, which enhances spatial prediction accuracy. Finally, in the decoder (bottom panel of Figure S8b), the model refines the activation regions from deep (Up_conv5) to shallow (Up_conv2) layers, focusing more precisely on areas of high *ACTB* expression. The final output layer (Conv_1 × 1) clearly identifies tissue regions with predominant *ACTB* expression, demonstrating HiST's ability to localize gene expression hotspots effectively during reconstruction.

### 2.4 Tumor Heterogeneity Depicted by HiST

Tumor heterogeneity is a key hallmark of cancer, with variations in DNA copy number, gene expression, and other molecular features across different tumor regions. Unlike single‐cell sequencing, which loses spatial context, ST preserves tissue architecture, thus revealing regional heterogeneity. Here, the potential of HiST‐reconstructed spatial GEPs to capture tumor spatial heterogeneity was evaluated. Using an ST slide from CRC as an example, HiST‐derived spatial GEPs within tumor regions were subjected to unsupervised clustering, revealing three distinct subclusters (C0, C1, C2; Figure 3f,g). Spatially, C1 and C2 were adjacent, with C2 primarily localized in the tumor core, while C1 was distributed around C2. On the other hand, C0 formed spatially independent clusters distinct from C1 and C2. Copy number variation (CNV) analysis was performed based on ground truth ST profiles to reveal the tumor heterogeneity. Spatial distribution of CNV scores was highly consistent with the spatial domains identified through unsupervised clustering of HiST‐predicted ST profiles (Figure 3h,i). These results, confirmed by high consistency with ground truth CNV patterns, demonstrate that HiST's molecular reconstruction successfully captures meaningful spatial patterns of tumor subclones and intra‐tumor heterogeneity from HE images. This demonstration serves as a proof‐of‐concept that HiST's high‐fidelity reconstruction yields reliable and interpretable biological signals for heterogeneity analysis, offering valuable insights for understanding tumor spatial biology.

### 2.5 HiST Accurately Predicts Patient Prognosis and Risk Stratification Through Pathological Image

**FIGURE 4:**
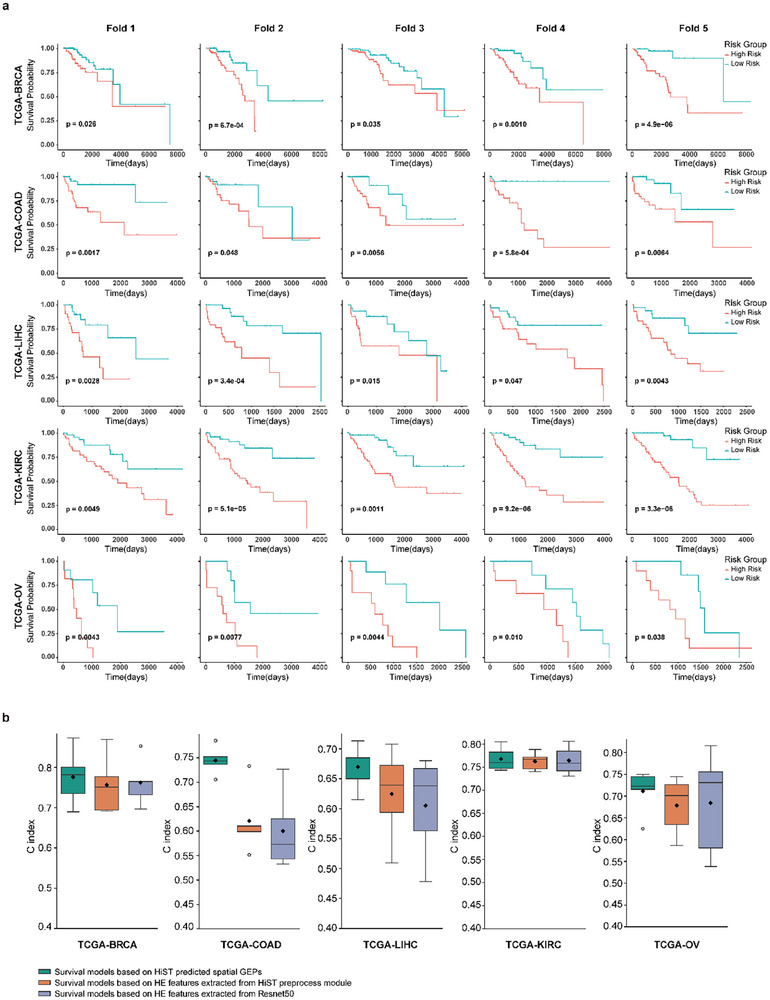
Prognosis performance of HiST survival models for five cancer types. a) Kaplan‐Meier plots of overall survival were generated according to the HiST predicted risk score for each validation fold of 5‐fold cross validation on five cancer type TCGA cohorts (p values were calculated by log‐rank test). b) 5‐fold cross‐validation performance comparison of different survival models in the five cancer cohorts of TCGA. In the boxplots, the minimum and maximum values of the C‐index are represented by horizontal lines, while the C‐index of the first quartile, median, and third quartile is represented by boxes. The diamond points represent the mean of the C‐index (*n* = 5), and the circular points represent outliers.

To further evaluate the clinical applicability of HiST, we employed HiST to infer spatial GEPs from HE‐stained histological images. Five cancer types from the TCGA database, each containing WSIs and survival data, were included: BRCA (*n* = 1,052), COAD (*n* = 434), LIHC (*n* = 341), KIRC (*n* = 512), and OV (*n* = 104) (Table S3). Prognostic predictions were performed using the HiST application module through 5‐fold cross‐validation. Risk scores were derived from HiST‐predicted spatial GEPs combined with clinical information for each validation fold. Across all five cancer types, patients classified as high‐risk (risk scores exceeding the median) showed significantly shorter overall survival compared to those in the low‐risk group (*p* < 0.05, log‐rank test; Figure 4a). These findings suggest that risk scores derived from HiST capture prognostic signals associated with patient outcomes.

To assess the predictive performance of HiST‐derived spatial GEPs, we compared it against HE‐stained image features extracted from pre‐trained Swin Transformer [30] used in HiST prediction module and features extracted from ResNet50 [43]. This comparison aims to validate whether the molecular features reconstructed by HiST provide superior prognostic value compared to the raw morphological features from which they were derived. Across all cancer types, survival models based on HiST‐predicted spatial GEPs outperformed those using HE‐stained image features (Figure 4b). For instance, in the TCGA‐COAD cohort, the HiST‐based survival model achieved an average concordance index (C‐index) of 0.74 in 5‐fold cross‐validation, significantly exceeding the performance of models using Swin Transformer (C‐index = 0.62) and ResNet50 (C‐index = 0.60). Similar results were observed in other cancer cohorts, including TCGA‐BRCA, where the HiST‐based survival model achieved a C‐index of 0.78. Overall, these results demonstrate that the high‐fidelity spatially molecular information reconstructed by HiST effectively predicts patient prognosis and enhances risk stratification compared to purely morphological features. The capacity of HiST to extract spatial GEPs from routine histological images suggests its utility as a reliable prognostic foundation for clinical decision support.

### 2.6 HiST Predicts Immunotherapy Outcomes and Reveals Molecular Differences Through Pathological Image

**FIGURE 5:**
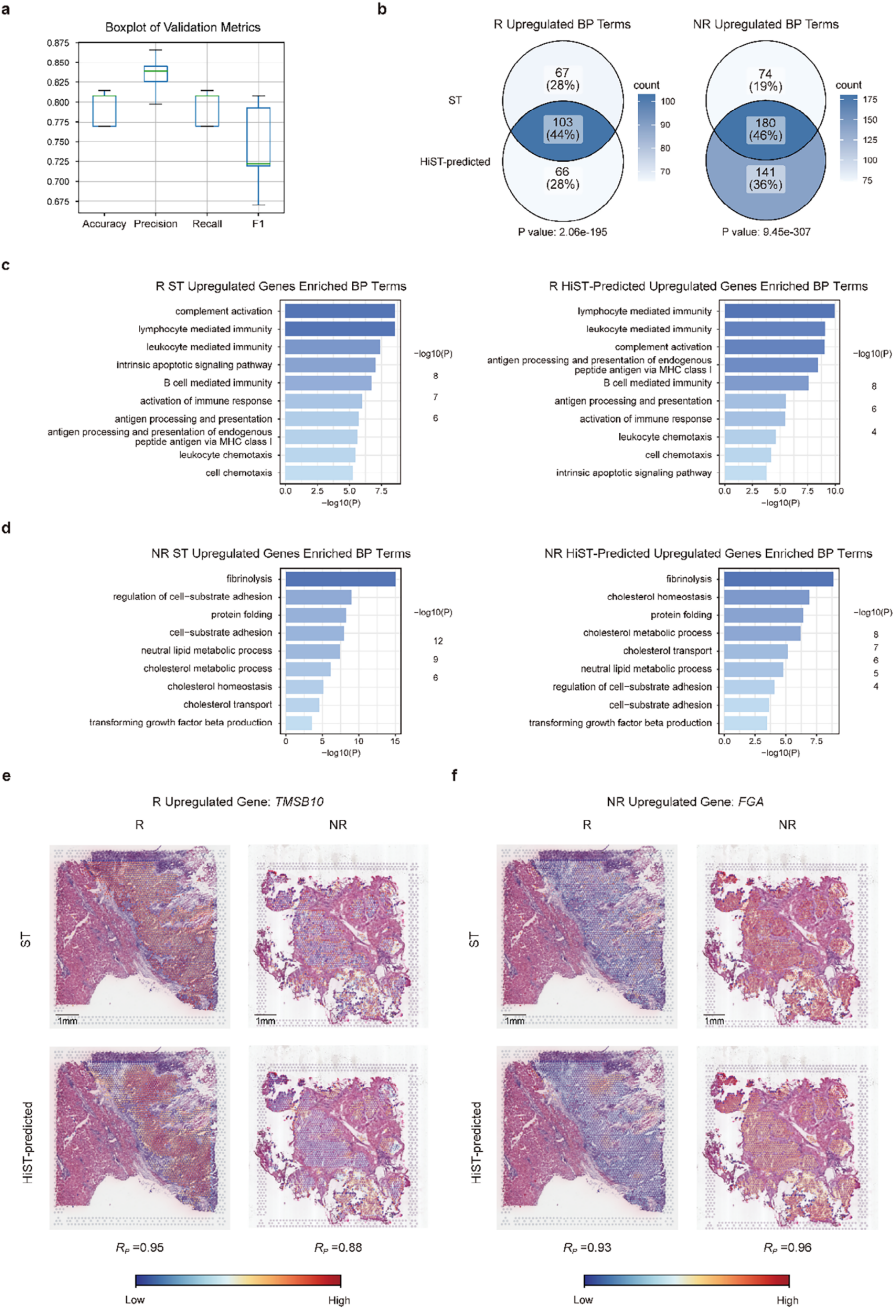
Prediction of outcomes and molecular characteristics of HCC patients after immunotherapy. a) Boxplot of ICB response prediction model evaluation metrics. b) Venn diagram of ST and HiST‐predicted upregulated genes enriched biological process (BP) terms for non‐response and response sample (p values were calculated by two‐tailed Fisher's exact test). c) Barplots of ST and HiST‐predicted upregulated genes enriched BP terms for responder (p values were calculated by hypergeometric test). d) Barplots of ST and HiST‐predicted upregulated genes enriched BP terms for non‐responder (p values were calculated by hypergeometric test). e) Visualization of ground truth and HiST‐predicted responder's upregulated gene *TMSB10* spatial gene expression (scale bar, 1 mm). f) Visualization of ground truth and HiST‐predicted non‐responder's upregulated gene *FGA* spatial gene expression (scale bar, 1 mm). ICB, immune checkpoint blockade; BP, biological process; R, responder; NR, non‐responder.

Previous studies have explored transcriptome‐based signatures, tumor mutation burden, and other omics‐level markers to predict patient response to immune checkpoint blockade (ICB) therapy [44, 45]. However, despite their widespread use in clinical diagnosis, HE‐stained images remain largely unexplored for evaluating immunotherapy efficacy. We applied HiST to predict immunotherapy outcomes using spatially resolved GEPs inferred from HE‐stained histology images to address this. A cohort of HCC patients who received immunotherapy was analyzed, consisting of WSIs along with corresponding clinical data and follow‐up records, including 29 responders (R) and 102 non‐responders (NR) (see Methods; Table S4). Using the HiST prediction module, spatially resolved GEPs were derived from HE‐stained images and treated as tissue‐level features. A 5‐fold cross‐validation was performed to assess HiST's ability to predict immunotherapy response, achieving an average accuracy of 0.79, precision of 0.85, recall of 0.79, and F1 score of 0.81 (Figure 5a). These results indicate that HiST‐predicted spatial GEPs capture signals associated with immunotherapy response.

To explore potential molecular differences related to immunotherapy response, spatial GEPs predicted by HiST were analyzed to compare the tumor microenvironment between responders and non‐responders. Spatial GEPs obtained from HiST predictions and the HCC 10X Visium dataset for both responders and non‐responders were analyzed. Spatially differentially expressed genes in tumor regions were identified by comparing the spatial GEPs of responders and non‐responders, followed by functional enrichment analysis. The enriched pathways from differentially expressed genes predicted by HiST showed a high degree of consistency with those identified through ST analysis. Specifically, 44% of the upregulated biological process (BP) terms in responders and 46% in non‐responders overlapped between these two spatial GEPs (Figure 5b). Both spatially differential GEPs revealed higher enrichment of immune activation‐related pathways in tumor regions of responders, including lymphocyte‐ and leukocyte‐mediated immunity, activation of the immune response, antigen processing and presentation [46], and leukocyte chemotaxis (Figure 5c). On the other hand, tumor regions of non‐responders had significant enrichment of pathways associated with immunotherapy resistance, including transforming growth factor beta production [47], cholesterol metabolic process, cholesterol transport [48], and cell‐substrate adhesion (Figure 5d). Further analysis of individual genes associated with immunotherapy efficacy revealed that thymosin beta‐10 (*TMSB10*) was significantly enriched in the tumor regions of responders compared to non‐responders. The *R_p_* between *TMSB10* spatial expression predicted by HiST and the ground truth from the ST dataset reached 0.88 in non‐responders and 0.95 in responders (Figure 5e). Similarly, the fibrinogen alpha chain (*FGA*) was significantly enriched in the tumor regions of non‐responders compared to responders. The *R_p_* between the HiST‐predicted spatial expression of *FGA* and the ground truth from the ST dataset reached 0.96 in non‐responders and 0.93 in responders (Figure 5f). These findings indicate that spatial GEPs predicted by HiST capture molecular differences consistent with ST data, supporting further investigation of immunotherapy‐related pathways.

## 3 Discussion

With advancements in artificial intelligence, ST technologies combined with paired HE‐stained histopathology images provide a unique opportunity to extract molecular information embedded within histological images. However, accurately predicting spatial GEPs from histological images remains a major challenge. Here, we introduce HiST, a novel framework that predicts spatial GEPs using cell morphology features in histological images. The performance of HiST was evaluated on 10X Visium datasets from five cancer types with 102 samples. The HiST tumor segmentation model achieved high accuracy in identifying tumor regions, with an AUC of 0.96 in breast cancer, comparable to annotations by expert pathologists. Comparison against existing models [8, 25, 26, 27] demonstrated HiST's robust performance in spatial GEP prediction. While existing methods achieve an average PCC of no more than 0.5, HiST significantly outperformed them with an average PCC of 0.74 across five cancer types. More importantly, HiST‐predicted spatial GEPs allowed effective prediction of cancer patient prognosis and immunotherapy outcomes, highlighting its potential as a powerful tool for clinical pathological diagnosis.

Previous advances in spatial GEP prediction primarily focused on incorporating spatial coordinate information into image‐processing workflows. However, methods that rely on absolute position embeddings struggle to effectively capture relative spatial relationships between patches, limiting their ability to model spatial context. Moreover, the high cost of acquiring ST data, combined with the large parameter sizes of mainstream deep learning models such as ViT and GNN, makes them prone to overfitting, particularly in small dataset scenarios. Compared to previous methods, HiST offers significant advantages in parameter efficiency, memory usage, and training speed (Figure 3c). These computational optimizations allow HiST to be conveniently trained and deployed on consumer‐grade personal computers, laying a solid foundation for its broader application in primary‐level clinical settings. HiST's better performance is due to its innovative combination of preprocessing techniques and highly efficient convolutional architecture. Specifically, cropping tissue regions surrounding the target spot allows the model to effectively use cell neighborhood information for gene prediction. Using pre‐trained models for image feature extraction reduces the learning burden on downstream prediction models, improving computational efficiency. Moreover, reconstructing spatial GEPs based on spatial coordinates preserves spatial context relationships, while segmentation‐based convolutional networks efficiently capture local features, learn the associations between gene spatial distribution and cell morphology, and use tissue boundaries to differentiate gene expression patterns, therefore achieving highly accurate predictions.

High‐fidelity spatial GEPs increase the applicability of HiST. HiST‐reconstructed spatial GEPs effectively distinguish tumor heterogeneity, allowing the identification of different tumor subclusters in histological images (Figure 3f–i). Moreover, HiST outperforms feature extraction methods based on Swin Transformer and ResNet50 in cancer prognosis prediction (Figure 4b). HiST is the first tool to apply spatial GEP prediction from histology to assess immunotherapy outcomes. The model's high accuracy, precision, recall rate, and F1 score highlight its robust performance in predicting patient responses to immunotherapy (Figure 5a). Furthermore, HiST‐predicted spatial GEPs are comparable to ground truth ST data, facilitating the identification of key genes and pathways involved in immunotherapy response regulation. These findings highlight HiST's potential application in personalized cancer treatment, offering novel insights into tumor biology and improving clinical decision‐making.

Due to the limitations of convolutional architectures in deep learning and the limited resolution of the training dataset, HiST requires fixed input and output sizes in the prediction and application modules. However, HiST pipeline incorporates a flexible image tiling and feature stitching mechanism that supports variable‐sized WSIs and diverse ST platforms. For results from other ST platforms (Figure S5a,b) and from TCGA whole‐slide images (Figure 3d,e), we applied blank padding to adjust the images to a 5:4 aspect ratio and then cropped them into 80×64 (5,120) square patches. Each patch was subsequently resized to the same sizes. The preprocessing module of HiST does not impose any constraints on the original resolution of the input images. For users who wish to obtain higher‐resolution HiST‐predicted ST profiles, an alternative approach is to divide the original image into N segments and process them in batches, thereby producing high‐resolution predictions with dimensions of gene number × (80×N) × (64×N).

Since downstream performance is strongly constrained by the quality of the upstream expression prediction, HiST achieves SOTA accuracy for the ST prediction based on HE images and provides a more reliable molecular representation for any subsequent analysis. HiST demonstrates significant clinical potential by predicting prognosis and immunotherapy response directly from routine HE images, offering a cost‐effective alternative to expensive ST. By reconstructing spatially resolved gene expression, HiST introduces a molecular layer to traditional histopathology, enabling a more detailed assessment of tumor heterogeneity and immune signatures. This approach complements existing biomarkers such as PD‐L1 staining and tumor mutational burden, providing spatially informed insights that may guide personalized treatment decisions. While further prospective validation is required, HiST's integration into digital pathology workflows could advance spatially informed precision oncology and enhance patient stratification in clinical practice.

The prediction of gene expression from HE images relies on the biological assumption that morphology reflects the underlying spatial arrangement of cell types and their associated marker genes. While HiST effectively captures spatially variable genes whose expression patterns align with histological morphology, this focus may introduce bias by favoring genes with strong spatial organization. However, many biologically or clinically relevant genes, such as signaling molecules, transcription factors, or early oncogenic drivers, exhibit subtle or diffuse expression patterns that are not visually discernible in histology. These genes remain challenging to predict from morphology alone, leading to lower confidence or stability in their inferred expression.

Another limitation of this study is the sample size imbalance between responders and non‐responders for immunotherapy. This imbalance could potentially bias the model toward predicting the group with a large sample size while reducing its sensitivity in correctly identifying smaller sample size groups. As a result, the model might exhibit a higher false negative rate for responders. Future studies with larger, more balanced cohorts will be essential for validating and improving the model's performance, particularly in better identifying smaller clinical subgroups. Additionally, techniques such as oversampling or adjusting the decision threshold could help address this issue and enhance model accuracy.

Moreover, current ST platforms, such as 10× Visium, capture only a limited number of spots (4,992 per slide), restricting spatial resolution and the diversity of detectable genes. The sparse and heterogeneous nature of most gene expression profiles further limits model generalization and hinders comprehensive tumor microenvironment analysis. Future advances in ST technologies and the availability of larger, higher‐resolution datasets will be critical to address these limitations. Integrating single‐cell transcriptomic references and multi‐modal data could extend model's predictive capacity beyond morphology‐linked genes, enabling more accurate and interpretable modeling of complex spatial gene expression landscapes across diverse cancer types.

## 4 Experimental Section

### 4.1 ST Data Preprocessing

The gene‐spot matrices generated from data processing from ST and Visium samples were processed using the R package Seurat (version 3.2.1) in R 4.3.1. Normalization of spot counts was performed using the LogVMR function.

### 4.2 Spatial Coordinates Transformation

The initial gene expression matrix was 2D, represented as *E* ^*B* × *G*^,  where rows (*B*) correspond to the barcodes of cells in the tissue region (maximum total number *n*  =  78 × 64), and columns (*G*) represent the set of all detected genes. HiST employs a cell segmentation model framework for regression model training; therefore, the gene expression matrix was transformed into a spatial expression map, where each channel corresponds to the spatial expression data of a candidate gene. Given that 10X Visium spots are hexagonally arranged rather than forming a rectangular grid, the spatial coordinates were transformed to construct a matrix while maintaining the relative spatial proximity of the spots. The spatial coordinates are defined as an ordered set of positions:

(1) $$P=\left\{\left(r_{i},c_{i},b_{i}\right)\right\}\;\begin{array}{c} n\\ i=1 \end{array}$$

where *r_i_* was the row index, *c_i_* was the column index, and *b_i_* represents the corresponding spot barcode. The spot ID matrix *S* ^78 × 64^ was populated according to the rule:

(2) $$S\;\left[r_{i}+1,\varphi \left(r_{i},c_{i}\right)\right]=b_{i}\;$$

where the mapping function φ(*r_i_*, *c_i_*) depends on the parity of the row index:

(3) $$\varphi \;\left(r_{i},c_{i}\right)=\left\{\begin{array}{c} \;\frac{c}{2}+1,if\;r_{i}\;\;is\;even\\ \frac{c+1}{2},if\;r_{i}\;\;is\;odd \end{array}\right.\;$$

### 4.3 Tumor Region Definition

In the tumor prediction task, tumor labels, serving as the training target, were inferred using spatial gene expression analysis with the R package Cottrazm (version 0.1.1). Within each tissue section, the correspondence between spot barcodes and tumor labels can be represented as:

(4) $$R=\left\{\left(b_{1},l_{1}\right),\left(b_{2},l_{2}\right),\text{…},\left(b_{n},l_{n}\right)\right\}\;$$

where *b_i_* denotes spot barcode and *l_i_* represents its corresponding tumor label. Then, through spatial coordinate transformation, this representation was converted into a tumor region mask *M* ^78 × 64^:

(5) $$M\;\left[r_{i}+1,\varphi \left(r_{i},c_{i}\right)\right]=\left\{\begin{array}{c} 1,\;if\;\;l_{i}\;\;is\;\;tumor\\ \;0,\;if\;\;l_{i}\;\;is\;\;normal \end{array}\right.\;$$

The ground truth used for training the tumor region prediction model was derived from Cottrazm‐BoundaryDefine, which infers tumor boundaries based on spatial transcriptomic molecular profiles. The annotations provided by the pathologist—based on histological image interpretation—were used independently as an auxiliary reference to qualitatively validate the accuracy of our model's predictions.

### 4.4 Target Genes Selection

ST enables the quantification of mRNA expression for a large number of genes within a specific region at a defined resolution. In 10× Visium, the number of genes detected through sequencing was significantly higher than the spatial coordinate dimension of the dataset. Gene selection was required to reduce the dimensionality of the target feature space to construct a spatial gene expression regression model using deep learning methods. Moran's I was a widely used statistical measure for detecting spatial autocorrelation in ST data, and it has been employed in multiple established ST analysis tools such as SPARK [49] and Seurat [50] to identify spatially variable genes. For each 10× Visium dataset, Moran's I and the corresponding *p*‐value were calculated for all detected genes across every sample within the dataset. Genes were selected as candidates if they met the following criteria: *p*‐value < 0.01 and median Moran's I > 0.4 across all samples. The Moran's I statistic, which measures spatial autocorrelation, was defined as:

(6) $$I=\frac{N}{S}\cdot \frac{\sum_{i=1}^{N}\sum_{j=1}^{N}w_{ij}\left(x_{i}-\bar{x}\right)\left(x_{j}-\bar{x}\right)}{\sum_{i=1}^{N}{\left(x_{i}-\bar{x}\right)}^{2}}$$

(7) $$S=\sum_{i=1}^{N}\sum_{j=1}^{N}w_{ij}\;$$

where *N* denotes the total number of spatial units, *x_i_* and *x_j_* represent the gene values for spatial units *i* and *j*, *w_ij_* was the spatial weight between units *i* and *j*, and *S* was the sum of all spatial weights. For spatial distance weight matrix construction, a weight of 1 was assigned to the 15 nearest neighbors, while all other weights were set to 0.

After selecting a subset *C* of genes from the set of all genes *G* as prediction candidates, spot barcodes were linked with their corresponding gene expression values through spatial coordinate transformation to generate a spatial expression map *M* ^78 × 64 × *C*^:

(8) $$M\;\left[r_{i}+1,\varphi \left(r_{i},c_{i}\right),g_{j}\right]=E_{ij}\;$$

where *g_j_* represents the selected gene candidate.

To further dissect the cellular composition of the tumor microenvironment and analyze cell regulatory networks, lineage markers of prevalent tumor cell types were integrated into the gene prediction targets for CRC and HCC. For the HER2 and cSCC datasets, gene sets from previously established spatial GEP prediction models [24, 25, 26] were used as predictive targets to ensure comprehensive evaluation. The precise number of target genes for each dataset was provided in Table S2.

### 4.5 Architecture of HiST

(a) Preprocess module:
(i) *HE image preprocessing*: Due to the heterogeneity in the specifications of HE images across different datasets, distinct methodologies were applied to segment HE images into patches according to the coordinates of the spots. Given that each sample from the 10× Visium technology contains 4,992 spots (78 × 64), the corresponding HE images were divided into 4,992 patches.

For all samples in the CRC 10X Visium dataset, including whole slide images, each patch was extracted to encompass both the spot and its surrounding tissue. The fiducial spot diameter was used as a reference, and a scale factor was applied to determine the side length of the patches, which were then resized to a resolution of 224 × 224 pixels.

In the BRCA, HCC, OV, and KIRC 10X Visium datasets, which contain only high‐resolution HE images with a resolution of approximately 2,000 × 2,000 pixels, 30 × 30 pixel regions surrounding the spots were extracted. For the HER2 and cSCC datasets, in which all samples contain whole slide images, the segmentation approach used in previous studies [26] was followed. HE images were divided into patches of 224 × 224 pixels based on the positions of the spots.

(ii) *Feature extraction*: HiST employs a segmentation‐based backbone network architecture to extract features from high‐resolution stained images and predict spot‐resolution information. The inherent resolution discrepancy between these images and spot‐level predictions can introduce misalignment during model training. To address this issue, CTranspath, a pre‐trained model integrating a convolutional neural network (CNN) with a multi‐scale Swin Transformer architecture, was used. This approach uses the robust feature representation capabilities of the pre‐trained model for stained images while simultaneously reducing the computational burden of training. HE images were partitioned into 4,992 patches, each of which was input into the CTranspath model. Before feature extraction, patches were resized to a resolution of 224 × 224 pixels and normalized channel‐wise using ImageNet‐derived mean and standard deviation values. The model processed each patch to generate a 768‐dimensional feature vector, resulting in a feature matrix with dimensions of 4,992 × 768.
(iii) *Feature map reconstruction*: To preserve the original spatial position information of the spots corresponding to the patches, the extracted features from each patch were concatenated into HE feature maps based on their respective positions within the HE image. This procedure ensured that each feature could be associated with its corresponding location on the original tissue section, retaining the spatial context information for each sample. To facilitate four down‐sampling operations during model processing, two extra features extracted from blank patches were appended to the height dimension of the feature map. Correspondingly, blank information was added to the target map, which included either the tumor region mask or the spatial gene expression map.
(b) *Prediction module*: The prediction module employs a modified U‐Net architecture designed to address the original U‐Net's limitations in capturing global context by incorporating skip connections. The overall structure of the encoder and decoder can be represented in Figure1b and the following equations:

(9) $$Encoder\;\left(X\right)=\left(DownSample\circ ConvBlock\circ ConvBlock\right)\left(X\right)$$

(10) $$Decoder\;\left(X\right)=\left(UpSample\circ ConvBlock\circ ConvBlock\right)\left(X\right)$$

The module processes HE feature maps of dimensions 768 × 80 × 64 as inputs, generating tumor segmentation masks (1 × 80 × 64) or gene expression maps (*gene* *num* × 80 × 64) as outputs. The model was implemented using the PyTorch package (Python 3.8.18, PyTorch 1.10.1+cu111).

The encoder consists of five convolutional levels, each comprising two convolution blocks and a down‐sampling operation. Each convolution block was composed of a convolution layer, batch normalization, and ReLU activation, which can be represented as:

(11) $$ConvBlock\left(X\right)=\left(ReLU\circ BN\circ Conv\right)\left(X\right)$$

where the convolution layer (nn.Conv2d) has a kernel size of 3 × 3, a stride of 1, and padding of 1. Down‐sampling was performed using max pooling with a 2 × 2 window. The number of output channels for the five convolutional levels was 64, 128, 256, 512, and 1,024.

The decoder was structurally symmetric to the encoder but with inverted input and output channel configurations. A multi‐scale attention gate connects the output from the first four convolutional levels to the decoder. The attention gate comprises three convolution layers with batch normalization: a pointwise convolution layer (nn.Conv2d with kernel size of 1 × 1), an ordinary convolution layer (nn.Conv2d with kernel size of 3 × 3, stride of 1 and padding of 1), and a dilated convolution layer (nn.Conv2d with kernel size of 3 × 3, stride of 1, padding of 2 and dilation rate of 2). The concatenated output was passed through a ReLU activation, followed by a pointwise convolution layer with batch normalization and sigmoid activation to filter features. The final level of convolutional feature maps was processed using a ConvMixer block, consisting of seven repeated combinations of depthwise convolution layers (nn.Conv2d with a kernel size of 7 × 7 and padding of 3) and pointwise convolution layers.

(c) *Application module*: The convolutional model ConvNeXt V2 was implemented to address downstream application tasks. The application module uses spatial GEPs predicted by the HiST prediction module as inputs, with risk scores or immunotherapy outcomes as outputs. The general model architecture consists of a stem block (nn.Conv2d with kernel size of 4 × 4 and stride of 4, followed by layer normalization), four stages of convolution blocks with down‐sampling layers (nn.Conv2d with a kernel size of 2×2 and stride of 2, preceded by layer normalization), global average pooling with layer normalization, and a linear layer (Figure 1). The ConvNeXt V2 block applies a series of operations, including depthwise convolution, pointwise convolutions, normalization, activation, and regularization (Figure S1). Let $X\in R^{N\times C\times H\times W}\;$ represent the input feature map, where *N* was the batch size, *C* was the number of channels, *H* was the height, and *W* was the width. The operations within the block can be described as follows:
(12) $$\begin{array}{ccc} & & Block\;\left(X\right)\\ & & \;=\left(DWConv\circ \mathrm{LN}\circ PWConv1\circ GELU\circ GRN\circ PWConv2\right)\\ & & \;\times \left(X\right)+DropPath\left(X\right) \end{array}$$

where *DWConv* represents a 2D depthwise convolutional layer with a kernel size of 7  × 7 and padding of 3; LN denotes Layer Normalization; *PWConv* was a pointwise (1 × 1) convolution, implemented as a linear layer; *GELU* represents the Gaussian Error Linear Unit activation function; *GRN* denotes the global response normalization module and *DropPath* represents the Drop Path regularization.

In the immunotherapy response prediction model, the convolution block features serve as input to a single linear layer that outputs the prediction. For survival analysis, the convolution block features are concatenated with one‐hot encoded clinical data (summarized in Table S3) and input into two additional linear layers within the head block.

### 4.6 Model Training Strategy

Different cross‐validation approaches were adopted depending on the dataset size and experimental objective in this study:

- For relatively small datasets used for benchmarking in tumor segmentation and spatial transcriptomic profile prediction tasks, including BRCA, CRC, HCC, KIRC, OV, HER2, and cSCC datasets (Table S1), we employed LOOCV as previous works. This strategy maximizes the use of limited samples and provides a robust assessment of generalizability across tissue sections.
- For experiments involving larger cohorts (TCGA datasets used in survival model and immune checkpoint blockade therapy HCC cohort used in immunotherapy response prediction model), we applied 5‐fold cross‐validation to ensure that both training and testing sets were sufficiently powered, while also controlling for patient‐level data leakage.

More specifically, in the prediction module, a LOOCV strategy was employed to evaluate the model's performance. The gene prediction model was trained using mean squared error (MSE) loss, while the tumor segmentation model was trained with binary cross‐entropy (BCE) loss. The training process employed the Adam optimizer with a weight decay of 10^−4^ and the CosineAnnealingLR learning rate scheduler, with an initial learning rate of 10^−3^. Training was conducted for up to 200 epochs with a batch size of 5.

A five‐fold cross‐validation approach was used to train and test in the application module. The AdamW optimizer with a momentum of 0.9 was applied for network optimization. The initial learning rate was set to 5 × 10^−3^, and training was performed for 300 epochs. For survival prediction, TCGA samples were randomly partitioned while maintaining the same proportion of censored and uncensored samples in each fold. The training set samples were grouped into a single batch for training using the Cox proportional hazards loss:

(13) $$loss=-\frac{1}{num_{uncensored}}\sum_{i=1}^{n}(risk_{i}-\log\left(\sum_{j=1}^{n}e^{risk_{j}}\right)\times censors_{i}$$

where *risk_i_* denotes the L2 normalized risk score for the *i_th_* observation, *risk_j_* represents the normalized L2 normalized risk score for the *j_th_* observation, and *censors_i_* represents the censoring indicator for the *i_th_* observation (1 for uncensored, 0 for censored). To ensure consistency across tumor types and align with common practice in related literature, we used overall survival as the prognostic outcome throughout our analyses.

The ICB prediction model was trained with cross‐entropy loss using the AdamW optimizer for 200 epochs with a batch size of 20. The initial learning rate was set to 5 × 10^−5^.

The specific hyperparameters for different training models are provided in Table S5. All other hyperparameters not listed in the table follow the default settings.

### 4.7 Benchmarking Scope and Methodology

We surveyed recent HE‐to‐transcriptomic prediction frameworks and evaluated their suitability for HE‐only spatial gene expression inference (Figure S7a). Models requiring ST inputs at inference were excluded. For all Visium‐based methods, models were trained using LOOCV for each dataset with default hyperparameters. SEQUOIA's k‐means aggregation module was removed to enable direct spot‐level prediction. iStar was trained following its standard single‐slide training protocol, and its cell‐level outputs were down‐sampled to spot‐level coordinates. For bulk‐level benchmarking, hist2RNA and SEQUOIA were trained to predict psedo‐bulk expression directly from WSI features. HiST's spot‐level predictions were aggregated to pseudo‐bulk values.

### 4.8 CNV Analysis

CNV scores were inferred using the R package inferCNV (v1.10.1). inferCNV infers large‐scale chromosomal copy number alterations from gene expression profiles and has been widely used to identify tumor subclones and intra‐tumoral heterogeneity. More specifically, we calculated CNV scores based on the real ST expression data, without incorporating any clustering information. Spots in the normal tissue region of the same slide were set as the reference group. For inferCNV‐run function, “HMM” was set to “True” to run hidden Markov model to predict CNV level and “tumor subcluster partition method” was set to “random_trees”.

### 4.9 Functional Enrichment Analysis

Differential expression analysis was performed using Scanpy (v1.10.3) rank_genes_groups function with default parameteres, applied independently to the ground truth ST profiles and the HiST‐predicted profiles. Differentially expressed genes (DEGs) were identified using a p‐value threshold of 0.01, as determined by the Student's t‐test. Enrichment analysis of upregulated and downregulated DEGs was performed separately using the clusterProfiler (v4.2.2).

### 4.10 Evaluation of Immunotherapy Efficacy in HCC Patients

The retrospective clinical cohort of 131 HCC patients in this study received immunotherapy and was collected from the First Affiliated Hospital of the University of Science and Technology of China. We retrieved patients' radiographic responses, which were used as the surrogate endpoint in this study, and conducted the assessment of treatment efficacy according to the Response Evaluation in Solid Tumors (RECIST v.1.1). The following measurements were used to evaluate therapy efficacy, patients with complete/partial response, or stable disease (SD) with more than six months of preogressive‐free survival were classified as responders, while patients with SD equal to or less than 6 months and progressive disease were categorized as non‐responders (classification annotated in Table S4).

### 4.11 Statistical Analysis

We employ Pearson and Spearman correlation coefficient to compare the predicted spatial gene expression with the observed gene expression to evaluate the model performance using Python (Python v3.8.18, scipy v1.10.1). Mean Pearson correlation coefficient across cross‐validation folds together with its 95% confidence interval was computed using the Student's t‐test.

In the survival model validation, patients in the validation set were stratified into high‐risk and low‐risk groups based on the median predicted risk score. To identify survival‐associated genes, predicted candidate genes and bulk RNA gene expression levels were each divided into high‐expression and low‐expression groups according to their respective median values, followed by log‐rank tests. The log‐rank test was conducted in Python (Python v3.8.18, lifelines v0.27.8) and the Kaplan‐Meier fitter was used to plot survival curves using R (R v4.3.1, survival v3.5.8, survminer v0.5.0).

## Author's Contribution

Y.Y. conceived and designed the project, Y.Y., Y.L, J.Z., and C.J. supervised the project. W.L., D.Z., C.J., and Y.Y. developed and implemented HiST, W.L., D.Z., E.P., and Y.Y. conducted all analyses. Y.L. and J.Z. conducted the clinical cohort and collected samples. Y.Z., J.L., and J.W. performed the experiment. W.L., D.Z., S.S., H.A.R., Y.L., J.Z., C.J., and Y.Y. were involved in the interpretation, W.L., D.Z., C.J., and Y.Y. wrote the manuscript.

## Conflicts of Interest

The authors declare no conflict of interest.

## Code Availability

The HiST software is available under the MIT License at https://github.com/Yelab2020/HiST, and a pre‐built Docker image is provided at https://hub.docker.com/r/bejsernia/hist/tags for convenient deployment.

## Supporting information




**Supporting File**: advs73517‐sup‐0001‐SuppMat.docx.

## Data Availability

The raw data of spatial transcriptomics and paired HE‐stained histological images with high resolution generated in this study were deposited in Genome Sequence Archive (GSA) with accession ID HRA006233 (The review link: https://ngdc.cncb.ac.cn/gsahuman/s/h2BCx7Cm). The HE‐stained histological images of 131 HCC tumor samples treated by immunotherapy were generated in this study were deposited at were deposited at Open Archive for Miscellaneous Data (OMIX) of National Genomics Data Center (NGDC) with accession ID OMIX009369 (https://ngdc.cncb.ac.cn/omix). Public spatial transcriptomics and paired HE‐stained histological images of 79 tumor tissues with covering five cancer types and with clear tumor boundaries were collected from the web available portal SpatialTME [51], including 42 samples from BRCA (10X genomics official website, https://www.10xgenomics.com/cn/resources/datasets, *n* = 4; Gene Expression Omnibus (GEO) with accession ID GSE210616, *n* = 35; GSE203612, *n* = 2; GSE176078, *n* = 1), CRC (GSA with accessible ID HRA000979, *n* = 2; in‐house data, *n* = 23), 13 samples from HCC (provided by Wu et al. [52], *n* = 6; provided by Liu et al. [53], *n* = 7), 14 samples from KIRC (provided by Meylan et al. [54], *n* = 14), 8 samples from OV (10X genomics official website, https://www.10xgenomics.com/cn/resources/datasets, *n* = 1; GSE211956, *n* = 5; GSE213699, *n* = 2), HER2 dataset (downloaded from https://github.com/almaan/her2st, *n* = 32), cSCC dataset (GSE144240, *n* = 12). Normalized gene expression data and paired HE‐stained histological images of BRCA (*n* = 1052), COAD (*n* = 434), LIHC (*n* = 341), KIRC (*n* = 512), and OV (*n* = 104) were downloaded from TCGA data portal (https://portal.gdc.cancer.gov).
