# Supplementary material for: HiST: Histological Images Reconstruct Tumor Spatial Transcriptomics via MultiScale Fusion Deep Learning
Source: Adv Sci (Weinh). 2026 Jan 5;13(13):e14351. doi: 10.1002/advs.202514351 (PMC12955878; doi:10.1002/advs.202514351)
Supplement: Supplementary file 1 — Supporting File: advs73517‐sup‐0001‐SuppMat.docx. [file ADVS-13-e14351-s001.docx]

Supporting Information

HiST: Histological Images Reconstruct Tumor Spatial Transcriptomics via MultiScale Fusion Deep Learning

*Wei Li*^#^*, Dong Zhang*^#^*, Eryu Peng, Shijun Shen,* *Hamid Alinejad-Rokny*, *Yao Liu*, Junke Zheng*, Cizhong Jiang*, Youqiong Ye**

^#^Co-first author

***Co-corresponding author

W. Li, S. Shen, C. Jiang

Shanghai Tenth People's Hospital, Shanghai Key Laboratory of Signaling and Disease Research, School of Life Sciences and Technology, Tongji University, Shanghai 200072, China.

E-mail: [czjiang@tongji.edu.cn](mailto:czjiang@tongji.edu.cn)

D. Zhang, E. Peng. Y. Ye

Shanghai Institute of Immunology, Department of Immunology and Microbiology, Shanghai Jiao Tong University School of Medicine, Shanghai 200025, China.

E-mail: [youqiong.ye@shsmu.edu.cn](mailto:youqiong.ye@shsmu.edu.cn)

Y. Liu

Department of Hepatobiliary Surgery, Centre for Leading Medicine and Advanced Technologies of IHM, The First Affiliated Hospital of USTC, Division of Life Sciences and Medicine, University of Science and Technology of China, Hefei, Anhui, 230001, China.

E-mail: liuyao66@ustc.edu.cn

J. Zheng

Institute for Translational Medicine on Cell Fate and Disease, Shanghai Ninth People's Hospital, Key Laboratory of Cell Differentiation and Apoptosis of National Ministry of Education, Department of Pathophysiology, Shanghai Jiao Tong University School of Medicine, Shanghai, China.

E-mail: [zhengjunke@shsmu.edu.cn](mailto:zhengjunke@shsmu.edu.cn)

H. Alinejad-Rokny

UNSW BioMedical Machine Learning Lab (BML) School of Biomedical Engineering, UNSW Sydney, Sydney, NSW 2052, Australia

**Supplementary Figures**


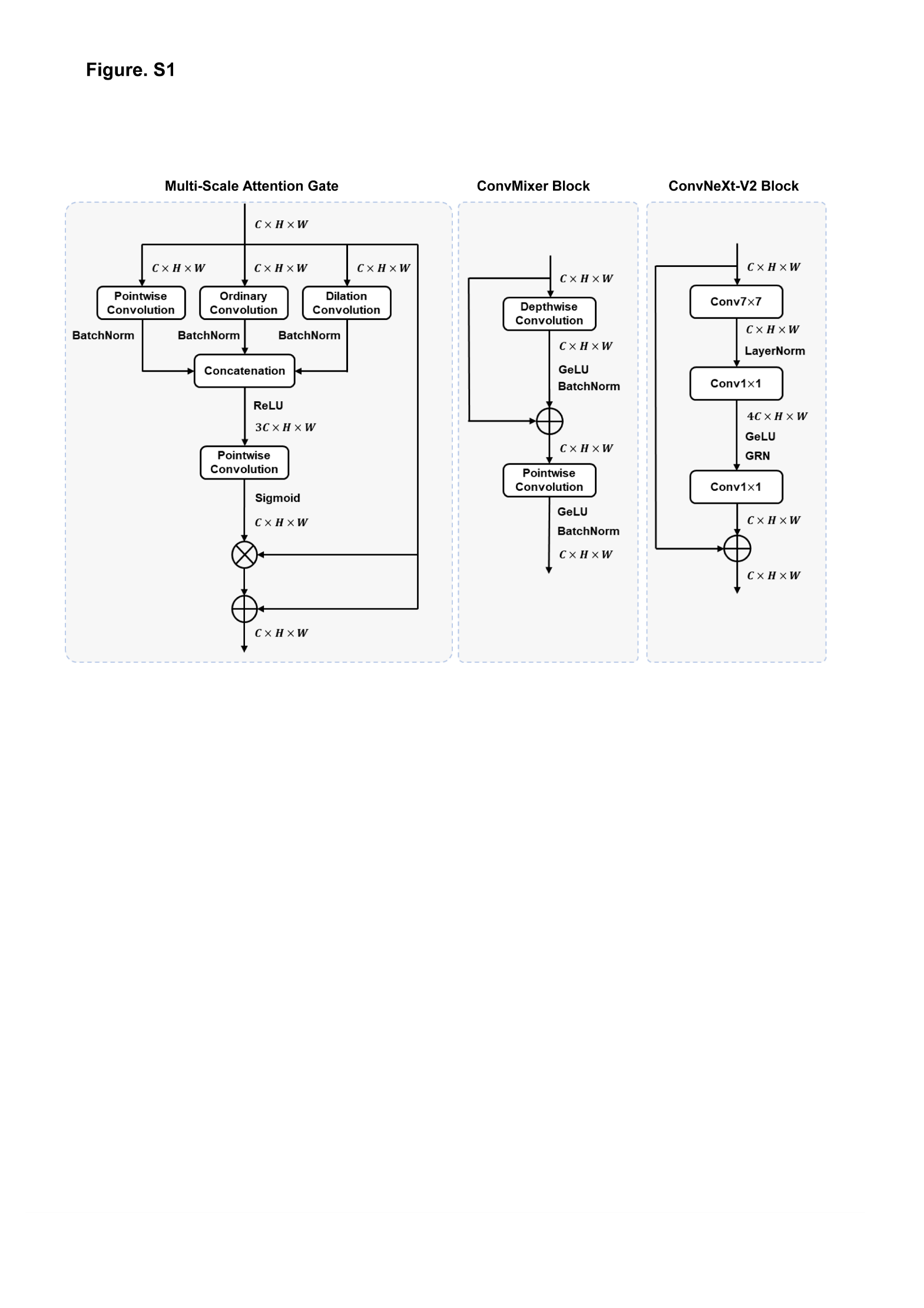


**Figure S1.** Architecture of three convolutional blocks in HiST. The framework of multi-scale attention gate, ConvMixer block, and ConvNeXt-V2 block which used in HiST.


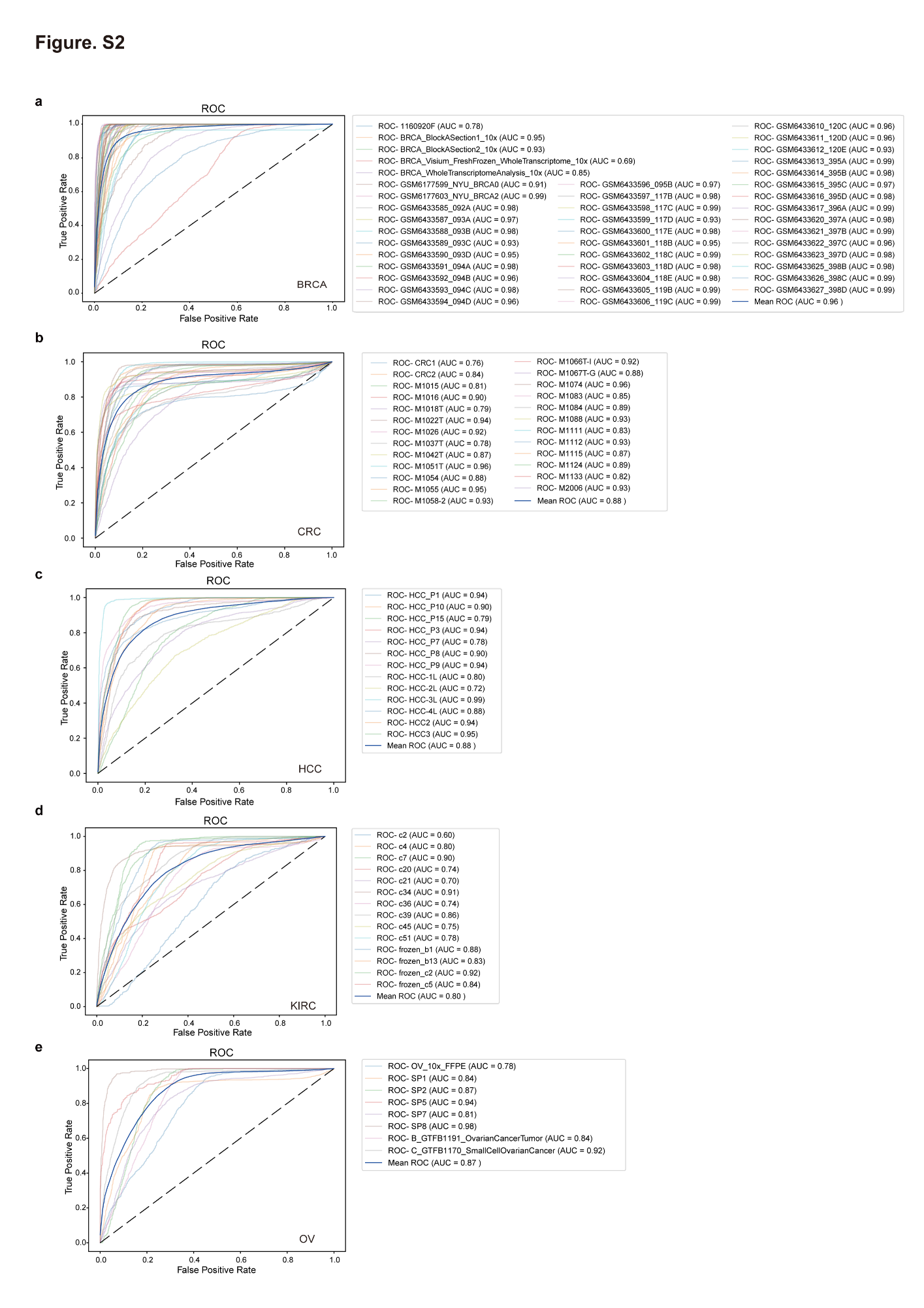


**Figure S2.** ROC curves of leave-one-out validation HiST tumor prediction models. **a-d)** ROC curve of leave-one-out validation tumor prediction models on BRCA dataset (*n* = 42, a), CRC dataset (*n* = 25, b). c, ROC curve of leave-one-out validation tumor prediction models on HCC dataset (*n* = 13, c), KIRC dataset (*n* = 14, d), and OV dataset (*n* = 8, e).


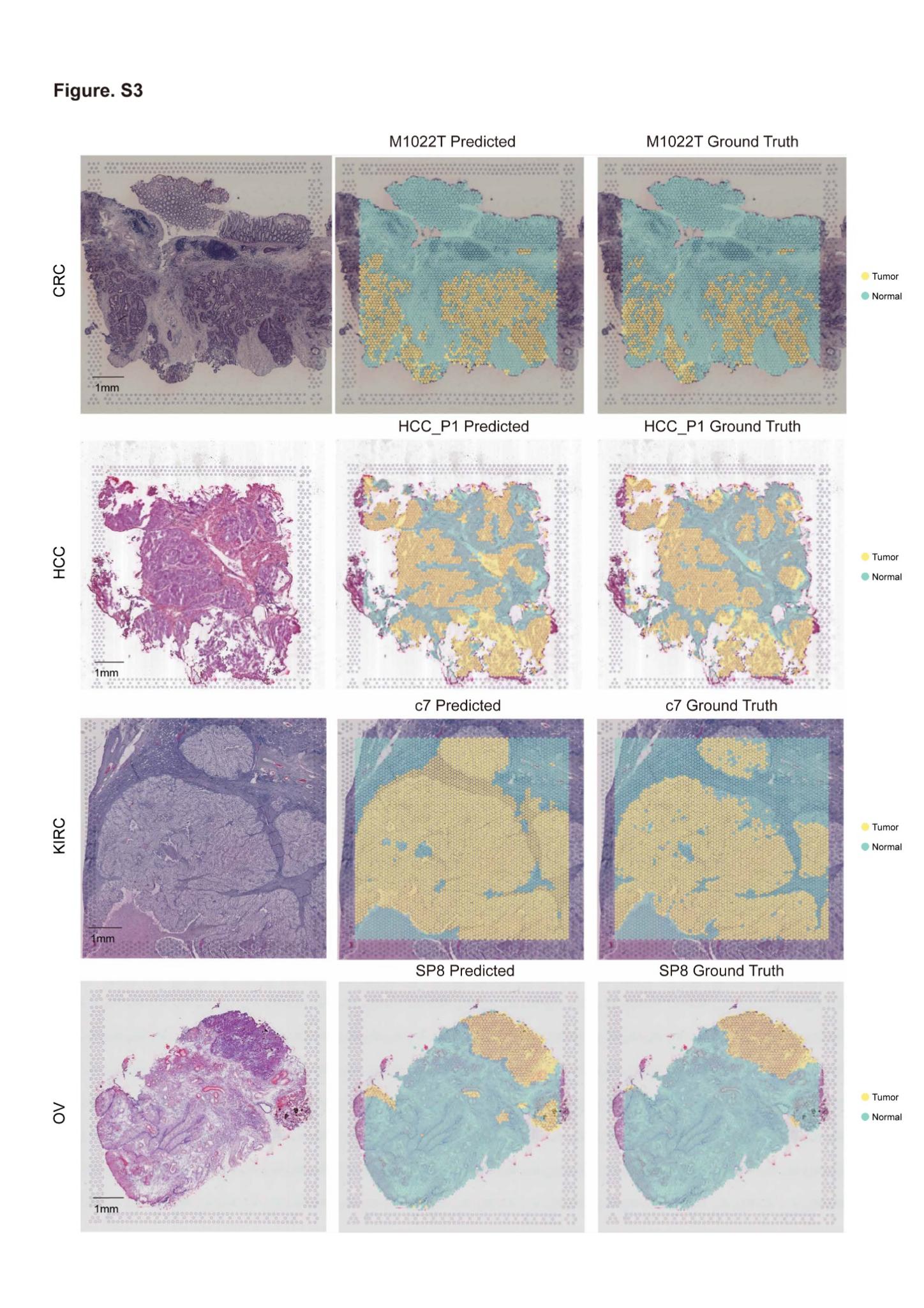


**Figure S3.** Visualization of ground truth and HiST-predicted tumor region. Visualization of ground truth and HiST-predicted tumor region on CRC, HCC, KIRC, and OV sample.


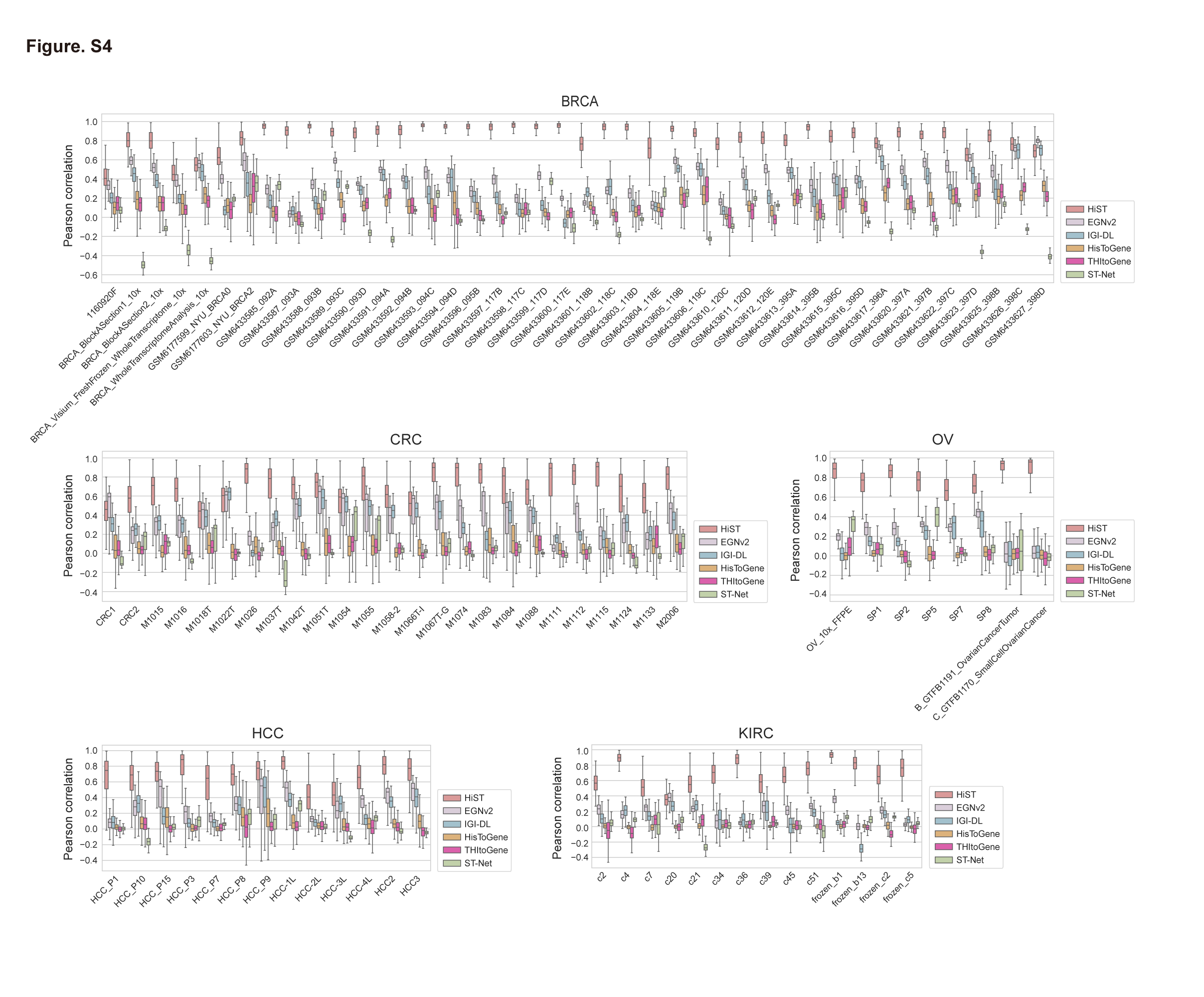


**Figure S4.** Leave-one-out validation Pearson correlation performance of spatial gene expression prediction methods. Boxplot showing Pearson correlation coefficient of 6 methods for spatial gene expression prediction on BRCA dataset (*n* = 42, a), CRC dataset (*n* = 25, b), OV dataset (*n* = 8, c), HCC dataset (*n* = 13, d), KIRC dataset (*n* = 14, e).


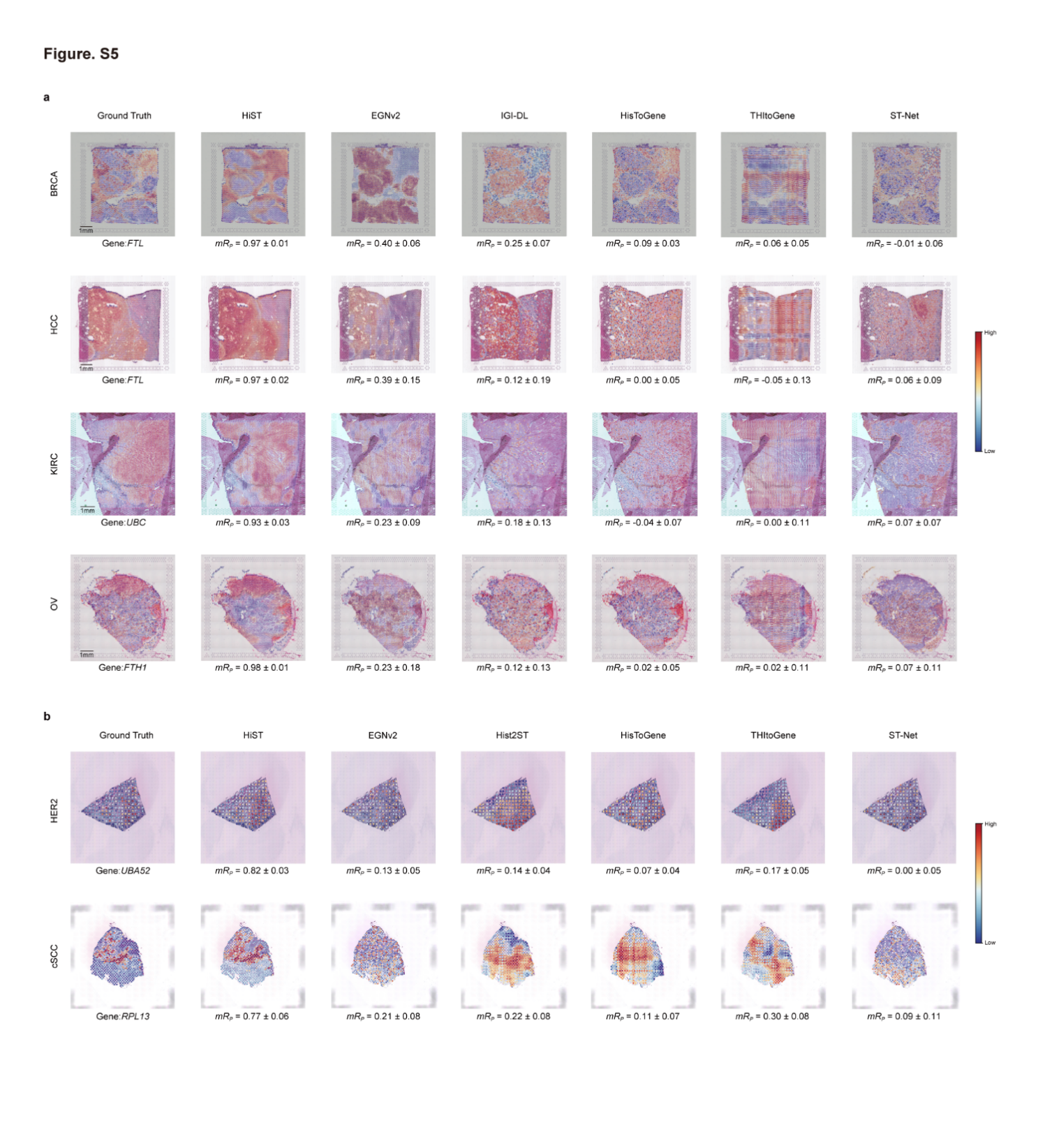


**Figure S5.** Spatial visualization of ground truth and predicted spatial gene expression. **a-b)** Spatial visualization of ground truth and predicted spatial gene expression of six models on four cancer types of 10X Visium samples (a) and HER2 and cSCC legacy ST dataset samples (b). Color bar indicates the gene expression level. *mRp* ± 95% CI denotes the mean Pearson correlation coefficient across cross-validation folds together with its 95% confidence interval computed using the Student’s t-test.


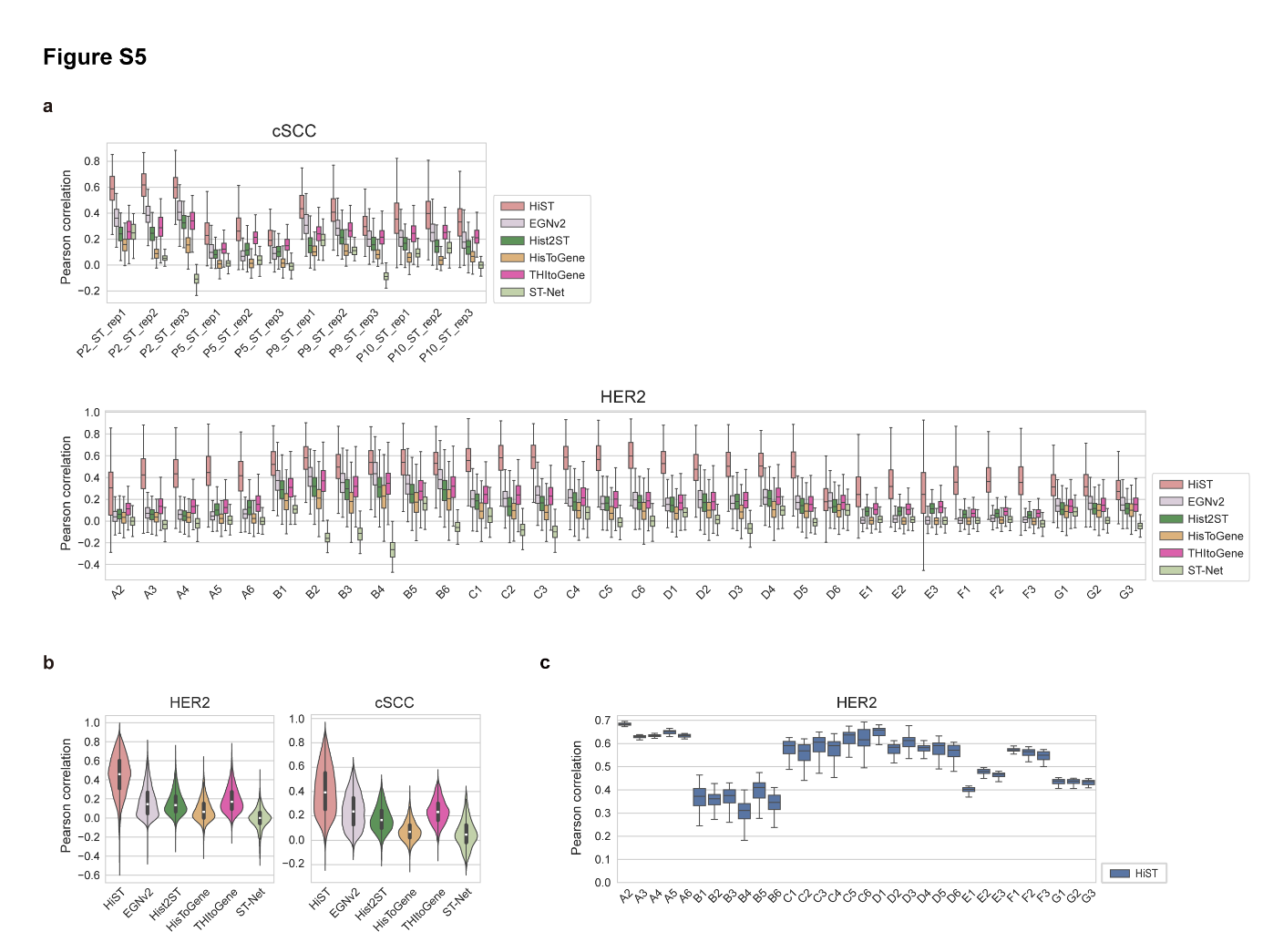


**Figure S6.** Performance evaluation of HiST in spatial gene expression prediction on legacy ST datasets. **a)** Boxplot showing Pearson correlation coefficient of 6 methods for spatial gene expression prediction on legacy ST cSCC dataset (*n* = 12), and HER2 dataset (*n* = 32). **b)** Viol in plots showing leave-one-out validation Pearson correlation performance of 6 models for spatial gene expression prediction on two legacy ST datasets. **c)** Cross-cohort generalization of HiST from BRCA 10X Visium to the HER2 legacy ST cohort. Forty-two models trained using BRCA Leave-one-out validation were evaluated on 32 HER2 slides; each box shows the mean Pearson correlation of all models for each slide, with one data point per model.


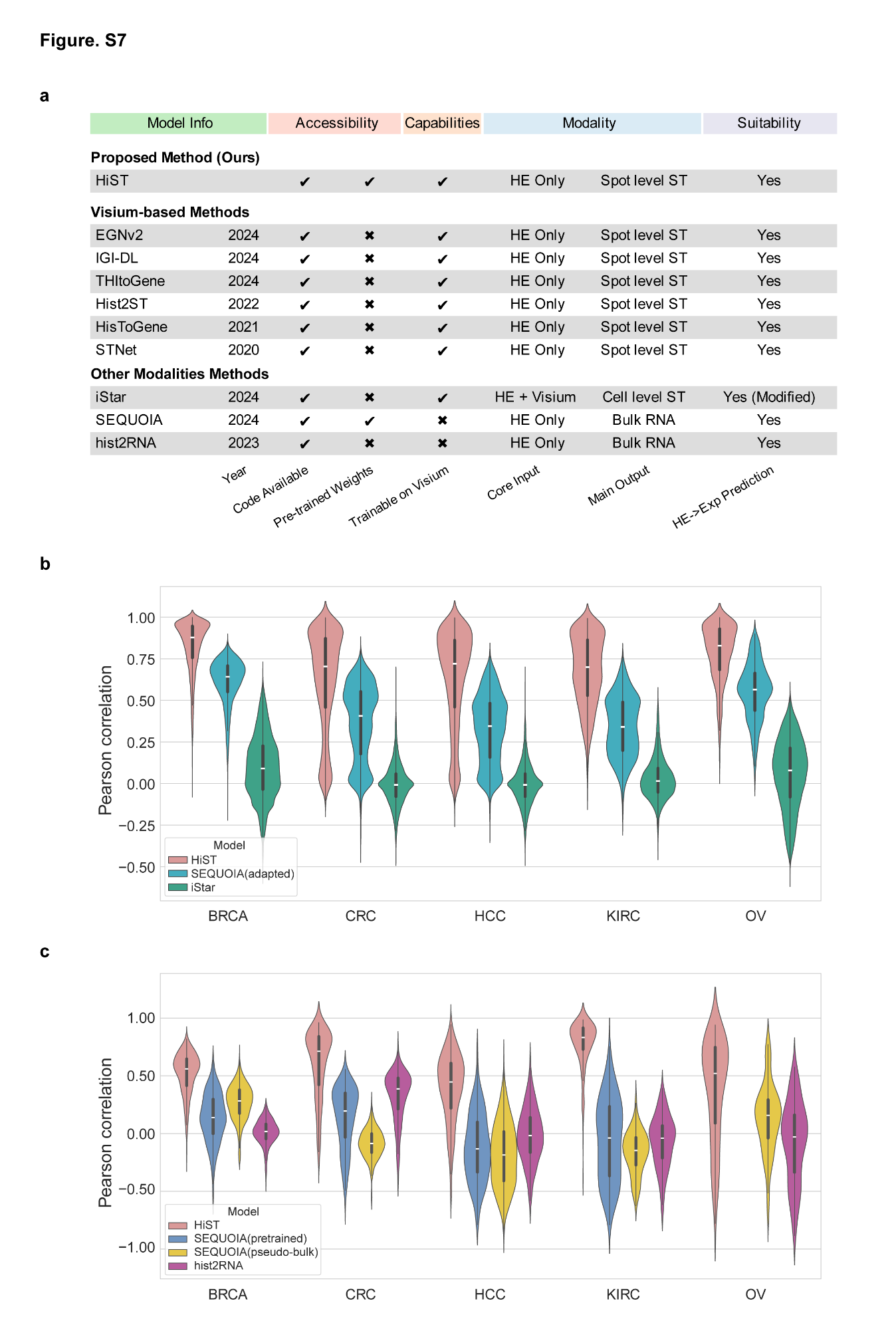


**Figure S7.** Comparative evaluation of Visium-based and alternative HE-to-transcriptomic prediction frameworks. **a)** Comparison of model capabilities and benchmarking suitability. This summary table positions HiST relative to the additional methods and previous baselines. Models are categorized based on their compatibility with the task of direct spot-level gene expression prediction from HE images. "Added Benchmarks" refers to methods integrated into the revised manuscript. "Excluded" refers to methods where direct comparison is infeasible due to fundamental architectural differences (*e.g*., requiring ST ground truth as input). "Core Input" highlights the dependency on specific data modalities (HE + Visium denotes methods requiring ST data during inference). "HE->Exp Prediction" indicates whether the model supports the primary task. **b)** Benchmarking HiST against other modality methods on spot-level. Violin plots display the Pearson Correlation Coefficient (PCC) of gene expression predictions across five cancer types. HiST and SEQUOIA (adapted) were evaluated using Leave-One-Out Cross-Validation (LOOCV); SEQUOIA was architecturally modified to remove k-means aggregation and retrained for direct patch-to-expression inference. iStar was evaluated by training on a single representative sample per dataset and validating on the remaining samples, with high-resolution outputs downsampled to match Visium spot coordinates. **c)** Benchmarking HiST against other modality methods on bulk-level. Benchmarking of sample-level predictive accuracy between HiST, SEQUOIA and hist2RNA using LOOCV. SEQUOIA (pretrained) was evaluated using the pretrained model weights released in the original study; weights for ovarian cancer were not provided. SEQUOIA (pseudo-bulk) and hist2RNA was trained to directly predict pseudo-bulk expression from WSI features. In contrast, HiST profiles were derived by aggregating fine-grained spot-level predictions into pseudo-bulk values.


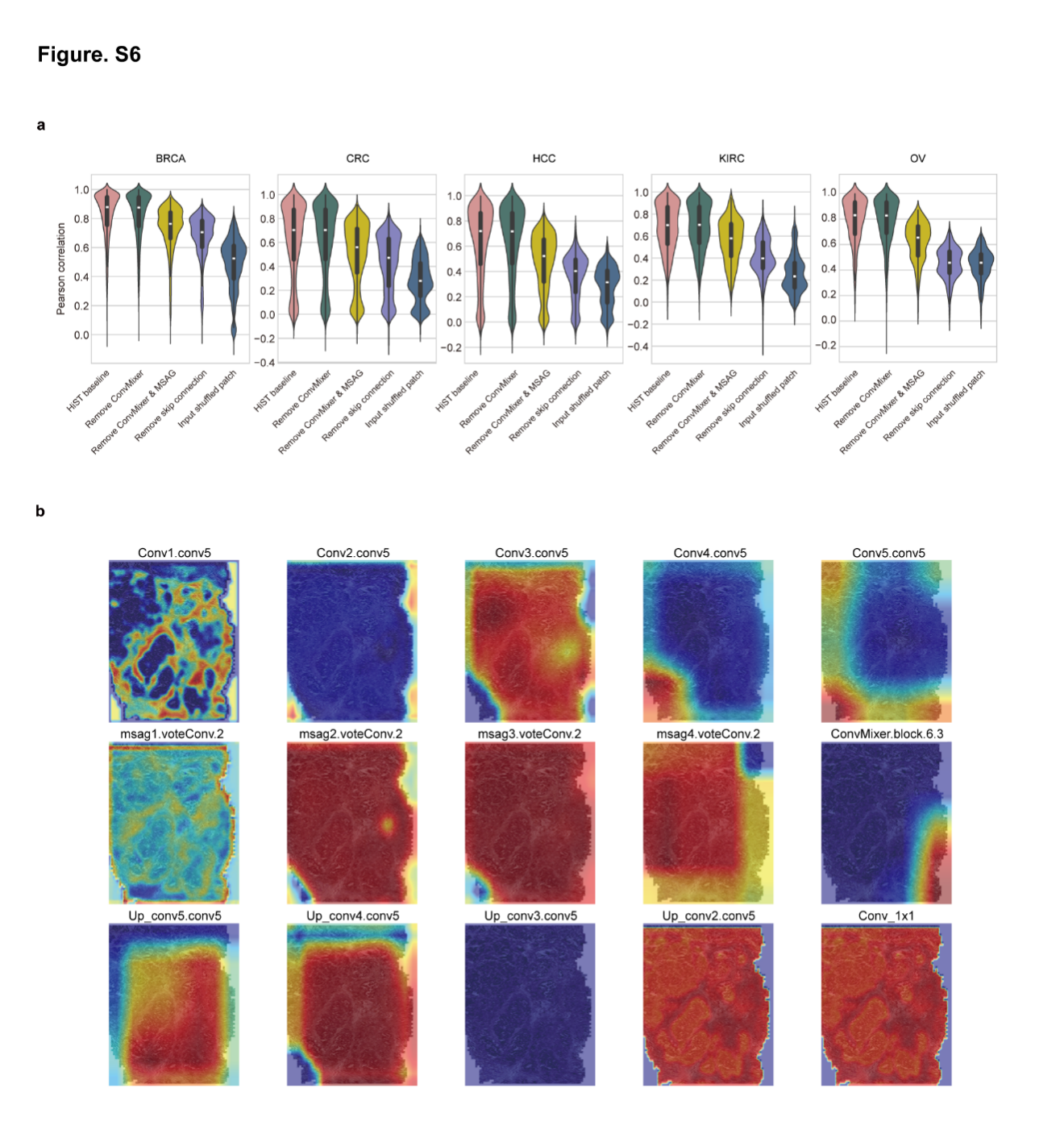


**Figure S8.** Ablation analysis and interpretability of HiST architecture. **a)** Comparison of HiST baseline and ablation variants in leave-one-out validation for spatial gene expression prediction on five 10X Visium ST datasets, including breast cancer (BRCA), colorectal cancer (CRC), hepatocellular carcinoma (HCC), kidney renal clear cell carcinoma (KIRC, and ovarian cancer (OV). Remove ConvMixer: replacing the ConvMixer blocks with direct connections; Remove ConvMixer & MSAG: replacing the ConvMixer blocks and multi-scale attention gate (MSAG) with direct connections; Remove skip connection: eliminating all skip connections; Input shuffled patch: randomizing the spatial arrangement of patches. **b)** Grad-CAM attribution maps from the last layer of each network module in HiST, illustrating hierarchical spatial features contributing to *ACTB* prediction.

**Supplementary Tables**

**Table S1.** Clinical characteristics of our training datasets.

| **Sample ID** | **Dataset** | **Cancers** | **Abbr.** | **Clinical description** | **Tissue type** | **Accessible ID** | **Published date** | **DOI** | **Platform** |
| --- | --- | --- | --- | --- | --- | --- | --- | --- | --- |
| OV_10x_FFPE | 10x | Ovarian cancer | OV | Serous papillary carcinoma of human ovarian | Primary tumor | 10x Visium Datasets |  | https://www.10xgenomics.com/cn/resources/datasets/human-ovarian-cancer-1-standard | Visium |
| GSM6506110_SP1 | GSE211956 | Ovarian cancer | OV | High-grade serous ovarian carcinoma (HGSOC),underwent taxane- and platinum-based neoadjuvant chemotherapy,Chemotherapy response score:1,which means poor response | Primary tumor | GSE211956 | 2022/8/29 | https://doi.org/10.1101/2022.08.29.505206 | Visium |
| GSM6506111_SP2 | GSE211956 | Ovarian cancer | OV | High-grade serous ovarian carcinoma (HGSOC),underwent taxane- and platinum-based neoadjuvant chemotherapy,Chemotherapy response score:3,which means good response | Primary tumor | GSE211956 | 2022/8/29 | https://doi.org/10.1101/2022.08.29.505206 | Visium |
| GSM6506114_SP5 | GSE211956 | Ovarian cancer | OV | High-grade serous ovarian carcinoma (HGSOC),underwent taxane- and platinum-based neoadjuvant chemotherapy,Chemotherapy response score:3,which means good response | Primary tumor | GSE211956 | 2022/8/29 | https://doi.org/10.1101/2022.08.29.505206 | Visium |
| GSM6506116_SP7 | GSE211956 | Ovarian cancer | OV | High-grade serous ovarian carcinoma (HGSOC),underwent taxane- and platinum-based neoadjuvant chemotherapy,Chemotherapy response score:1,which means poor response | Primary tumor | GSE211956 | 2022/8/29 | https://doi.org/10.1101/2022.08.29.505206 | Visium |
| GSM6506117_SP8 | GSE211956 | Ovarian cancer | OV | High-grade serous ovarian carcinoma (HGSOC),underwent taxane- and platinum-based neoadjuvant chemotherapy,Chemotherapy response score:1,which means poor response | Primary tumor | GSE211956 | 2022/8/29 | https://doi.org/10.1101/2022.08.29.505206 | Visium |
| B_GTFB1191_OvarianCancerTumor | GSE213699 | Ovarian cancer | OV | High-grade serous ovarian carcinoma (HGSOC),primary Tumor (pT): pT3c: Macroscopic peritoneal metastasis beyond pelvis more than 2 cm in greatest dimension with or without metastasis to the retroperitoneal lymph nodes (includes extension of tumor to capsule of liver and spleen without parenchymal involvement of either organ); Regional Lymph Nodes (pN): pN1b: Metastasis more than 10 mm in greatest dimension; FIGO Stage (2015 FIGO Cancer Report): IIIC: Macroscopic peritoneal metastases beyond the pelvic brim >2 cm in greatest dimension, with or without metastases to the retroperitoneal nodes | Primary tumor | GSE213699 | 2022/10/3 | https://doi.org/10.1016/j.gore.2022.101077 | Visium |
| C_GTFB1170_SmallCellOvarianCancer | GSE213699 | Ovarian cancer | OV | Small cell carcinoma of the ovary hypercalcemic type (SCCOHT),Primary Tumor (pT): pT1a: Tumor limited to one ovary (capsule intact) or fallopian tube, no tumor on ovarian or fallopian tube surface; no malignant cells in ascites or peritoneal washings; Regional Lymph Nodes (pN): pN1b: Metastasis more than 10 mm in greatest dimension; FIGO Stage (2015 FIGO Cancer Report): IIIA1(ii): Metastasis >10 mm in greatest dimension | Primary tumor | GSE213699 | 2022/10/3 | https://doi.org/10.1016/j.gore.2022.101077 | Visium |
| c2 | Pmid: 35231421 | Renal cell carcinoma | RCC | Clear cell renal cell carcinoma | Primary tumor | Pmid: 35231421 | 2022/3/8 | https://doi.org/10.1016/j.immuni.2022.02.001 | Visium |
| c4 | Pmid: 35231421 | Renal cell carcinoma | RCC | Clear cell renal cell carcinoma | Primary tumor | Pmid: 35231421 | 2022/3/8 | https://doi.org/10.1016/j.immuni.2022.02.001 | Visium |
| c7 | Pmid: 35231421 | Renal cell carcinoma | RCC | Clear cell renal cell carcinoma | Primary tumor | Pmid: 35231421 | 2022/3/8 | https://doi.org/10.1016/j.immuni.2022.02.001 | Visium |
| c20 | Pmid: 35231421 | Renal cell carcinoma | RCC | Clear cell renal cell carcinoma | Primary tumor | Pmid: 35231421 | 2022/3/8 | https://doi.org/10.1016/j.immuni.2022.02.001 | Visium |
| c21 | Pmid: 35231421 | Renal cell carcinoma | RCC | Clear cell renal cell carcinoma | Primary tumor | Pmid: 35231421 | 2022/3/8 | https://doi.org/10.1016/j.immuni.2022.02.001 | Visium |
| c34 | Pmid: 35231421 | Renal cell carcinoma | RCC | Clear cell renal cell carcinoma | Primary tumor | Pmid: 35231421 | 2022/3/8 | https://doi.org/10.1016/j.immuni.2022.02.001 | Visium |
| c36 | Pmid: 35231421 | Renal cell carcinoma | RCC | Clear cell renal cell carcinoma | Primary tumor | Pmid: 35231421 | 2022/3/8 | https://doi.org/10.1016/j.immuni.2022.02.001 | Visium |
| c39 | Pmid: 35231421 | Renal cell carcinoma | RCC | Clear cell renal cell carcinoma | Primary tumor | Pmid: 35231421 | 2022/3/8 | https://doi.org/10.1016/j.immuni.2022.02.001 | Visium |
| c45 | Pmid: 35231421 | Renal cell carcinoma | RCC | Clear cell renal cell carcinoma | Primary tumor | Pmid: 35231421 | 2022/3/8 | https://doi.org/10.1016/j.immuni.2022.02.001 | Visium |
| c51 | Pmid: 35231421 | Renal cell carcinoma | RCC | Clear cell renal cell carcinoma | Primary tumor | Pmid: 35231421 | 2022/3/8 | https://doi.org/10.1016/j.immuni.2022.02.001 | Visium |
| frozen_b1 | Pmid: 35231421 | Renal cell carcinoma | RCC | Clear cell renal cell carcinoma | Primary tumor | Pmid: 35231421 | 2022/3/8 | https://doi.org/10.1016/j.immuni.2022.02.001 | Visium |
| frozen_b13 | Pmid: 35231421 | Renal cell carcinoma | RCC | Clear cell renal cell carcinoma | Primary tumor | Pmid: 35231421 | 2022/3/8 | https://doi.org/10.1016/j.immuni.2022.02.001 | Visium |
| frozen_c2 | Pmid: 35231421 | Renal cell carcinoma | RCC | Clear cell renal cell carcinoma | Primary tumor | Pmid: 35231421 | 2022/3/8 | https://doi.org/10.1016/j.immuni.2022.02.001 | Visium |
| frozen_c5 | Pmid: 35231421 | Renal cell carcinoma | RCC | Clear cell renal cell carcinoma | Primary tumor | Pmid: 35231421 | 2022/3/8 | https://doi.org/10.1016/j.immuni.2022.02.001 | Visium |
| BRCA(Breast cancer)_BlockASection1_10x | 10x | Breast cancer | BRCA | IDC,fresh frozen Invasive Ductal Carcinoma breast tissue. AJCC/UICC Stage Group IIA, ER positive, PR negative, Her2 positive, annotate:Ductal carcinoma in situ, Lobular carcinoma in situ, Invasive Carcinoma | Primary tumor | 10x Visium Datasets |  | https://www.10xgenomics.com/cn/resources/datasets/human-breast-cancer-block-a-section-1-1-standard-1-1-0 | Visium |
| BRCA(Breast cancer)_BlockASection2_10x | 10x | Breast cancer | BRCA | IDC,fresh frozen Invasive Ductal Carcinoma breast tissue. AJCC/UICC Stage Group IIA, ER positive, PR negative, Her2 positive, annotate:Ductal carcinoma in situ, Lobular carcinoma in situ, Invasive Carcinoma | Primary tumor | 10x Visium Datasets |  | https://www.10xgenomics.com/cn/resources/datasets/human-breast-cancer-block-a-section-2-1-standard-1-1-0 | Visium |
| BRCA(Breast cancer)_Visium_FreshFrozen_WholeTranscriptome_10x | 10x | Breast cancer | BRCA | IDC,fresh frozen Invasive Ductal Carcinoma breast tissue. AJCC/UICC Stage T2N0M0, ER positive, PR negative, Hercep Test 2+. | Primary tumor | 10x Visium Datasets |  | https://www.10xgenomics.com/cn/resources/datasets/human-breast-cancer-visium-fresh-frozen-whole-transcriptome-1-standard | Visium |
| BRCA(Breast cancer)_WholeTranscriptomeAnalysis_10x | 10x | Breast cancer | BRCA | ILC,fresh frozen human Invasive Lobular Carcinoma breast tissue. AJCC/UICC Stage Group I, ER positive, PR positive, HER2 negative. | Primary tumor | 10x Visium Datasets |  | https://www.10xgenomics.com/cn/resources/datasets/human-breast-cancer-whole-transcriptome-analysis-1-standard-1-2-0 | Visium |
| 1160920F | GSE176078 | Breast cancer | BRCA | TNBC | Primary tumor | GSE176078 | 2021/9/6 | https://doi.org/10.1038/s41588-021-00911-1 | Visium |
| GSM6433585_092A | GSE210616 | Breast cancer | BRCA | TNBC | Primary tumor | GSE210616 | 2023/1/4 | https://doi.org/10.1158/0008-5472.can-22-2682 | Visium |
| GSM6433587_093A | GSE210616 | Breast cancer | BRCA | TNBC | Primary tumor | GSE210616 | 2023/1/4 | https://doi.org/10.1158/0008-5472.can-22-2682 | Visium |
| GSM6433588_093B | GSE210616 | Breast cancer | BRCA | TNBC | Primary tumor | GSE210616 | 2023/1/4 | https://doi.org/10.1158/0008-5472.can-22-2682 | Visium |
| GSM6433589_093C | GSE210616 | Breast cancer | BRCA | TNBC | Primary tumor | GSE210616 | 2023/1/4 | https://doi.org/10.1158/0008-5472.can-22-2682 | Visium |
| GSM6433590_093D | GSE210616 | Breast cancer | BRCA | TNBC | Primary tumor | GSE210616 | 2023/1/4 | https://doi.org/10.1158/0008-5472.can-22-2682 | Visium |
| GSM6433591_094A | GSE210616 | Breast cancer | BRCA | TNBC | Primary tumor | GSE210616 | 2023/1/4 | https://doi.org/10.1158/0008-5472.can-22-2682 | Visium |
| GSM6433592_094B | GSE210616 | Breast cancer | BRCA | TNBC | Primary tumor | GSE210616 | 2023/1/4 | https://doi.org/10.1158/0008-5472.can-22-2682 | Visium |
| GSM6433593_094C | GSE210616 | Breast cancer | BRCA | TNBC | Primary tumor | GSE210616 | 2023/1/4 | https://doi.org/10.1158/0008-5472.can-22-2682 | Visium |
| GSM6433594_094D | GSE210616 | Breast cancer | BRCA | TNBC | Primary tumor | GSE210616 | 2023/1/4 | https://doi.org/10.1158/0008-5472.can-22-2682 | Visium |
| GSM6433596_095B | GSE210616 | Breast cancer | BRCA | TNBC | Primary tumor | GSE210616 | 2023/1/4 | https://doi.org/10.1158/0008-5472.can-22-2682 | Visium |
| GSM6433597_117B | GSE210616 | Breast cancer | BRCA | TNBC | Primary tumor | GSE210616 | 2023/1/4 | https://doi.org/10.1158/0008-5472.can-22-2682 | Visium |
| GSM6433598_117C | GSE210616 | Breast cancer | BRCA | TNBC | Primary tumor | GSE210616 | 2023/1/4 | https://doi.org/10.1158/0008-5472.can-22-2682 | Visium |
| GSM6433599_117D | GSE210616 | Breast cancer | BRCA | TNBC | Primary tumor | GSE210616 | 2023/1/4 | https://doi.org/10.1158/0008-5472.can-22-2682 | Visium |
| GSM6433600_117E | GSE210616 | Breast cancer | BRCA | TNBC | Primary tumor | GSE210616 | 2023/1/4 | https://doi.org/10.1158/0008-5472.can-22-2682 | Visium |
| GSM6433601_118B | GSE210616 | Breast cancer | BRCA | TNBC | Primary tumor | GSE210616 | 2023/1/4 | https://doi.org/10.1158/0008-5472.can-22-2682 | Visium |
| GSM6433602_118C | GSE210616 | Breast cancer | BRCA | TNBC | Primary tumor | GSE210616 | 2023/1/4 | https://doi.org/10.1158/0008-5472.can-22-2682 | Visium |
| GSM6433603_118D | GSE210616 | Breast cancer | BRCA | TNBC | Primary tumor | GSE210616 | 2023/1/4 | https://doi.org/10.1158/0008-5472.can-22-2682 | Visium |
| GSM6433604_118E | GSE210616 | Breast cancer | BRCA | TNBC | Primary tumor | GSE210616 | 2023/1/4 | https://doi.org/10.1158/0008-5472.can-22-2682 | Visium |
| GSM6433605_119B | GSE210616 | Breast cancer | BRCA | TNBC | Primary tumor | GSE210616 | 2023/1/4 | https://doi.org/10.1158/0008-5472.can-22-2682 | Visium |
| GSM6433606_119C | GSE210616 | Breast cancer | BRCA | TNBC | Primary tumor | GSE210616 | 2023/1/4 | https://doi.org/10.1158/0008-5472.can-22-2682 | Visium |
| GSM6433610_120C | GSE210616 | Breast cancer | BRCA | TNBC | Primary tumor | GSE210616 | 2023/1/4 | https://doi.org/10.1158/0008-5472.can-22-2682 | Visium |
| GSM6433611_120D | GSE210616 | Breast cancer | BRCA | TNBC | Primary tumor | GSE210616 | 2023/1/4 | https://doi.org/10.1158/0008-5472.can-22-2682 | Visium |
| GSM6433612_120E | GSE210616 | Breast cancer | BRCA | TNBC | Primary tumor | GSE210616 | 2023/1/4 | https://doi.org/10.1158/0008-5472.can-22-2682 | Visium |
| GSM6433613_395A | GSE210616 | Breast cancer | BRCA | TNBC | Primary tumor | GSE210616 | 2023/1/4 | https://doi.org/10.1158/0008-5472.can-22-2682 | Visium |
| GSM6433614_395B | GSE210616 | Breast cancer | BRCA | TNBC | Primary tumor | GSE210616 | 2023/1/4 | https://doi.org/10.1158/0008-5472.can-22-2682 | Visium |
| GSM6433615_395C | GSE210616 | Breast cancer | BRCA | TNBC | Primary tumor | GSE210616 | 2023/1/4 | https://doi.org/10.1158/0008-5472.can-22-2682 | Visium |
| GSM6433616_395D | GSE210616 | Breast cancer | BRCA | TNBC | Primary tumor | GSE210616 | 2023/1/4 | https://doi.org/10.1158/0008-5472.can-22-2682 | Visium |
| GSM6433617_396A | GSE210616 | Breast cancer | BRCA | TNBC | Primary tumor | GSE210616 | 2023/1/4 | https://doi.org/10.1158/0008-5472.can-22-2682 | Visium |
| GSM6433620_397A | GSE210616 | Breast cancer | BRCA | TNBC | Primary tumor | GSE210616 | 2023/1/4 | https://doi.org/10.1158/0008-5472.can-22-2682 | Visium |
| GSM6433621_397B | GSE210616 | Breast cancer | BRCA | TNBC | Primary tumor | GSE210616 | 2023/1/4 | https://doi.org/10.1158/0008-5472.can-22-2682 | Visium |
| GSM6433622_397C | GSE210616 | Breast cancer | BRCA | TNBC | Primary tumor | GSE210616 | 2023/1/4 | https://doi.org/10.1158/0008-5472.can-22-2682 | Visium |
| GSM6433623_397D | GSE210616 | Breast cancer | BRCA | TNBC | Primary tumor | GSE210616 | 2023/1/4 | https://doi.org/10.1158/0008-5472.can-22-2682 | Visium |
| GSM6433625_398B | GSE210616 | Breast cancer | BRCA | TNBC | Primary tumor | GSE210616 | 2023/1/4 | https://doi.org/10.1158/0008-5472.can-22-2682 | Visium |
| GSM6433626_398C | GSE210616 | Breast cancer | BRCA | TNBC | Primary tumor | GSE210616 | 2023/1/4 | https://doi.org/10.1158/0008-5472.can-22-2682 | Visium |
| GSM6433627_398D | GSE210616 | Breast cancer | BRCA | TNBC | Primary tumor | GSE210616 | 2023/1/4 | https://doi.org/10.1158/0008-5472.can-22-2682 | Visium |
| GSM6177599_NYU_BRCA(Breast cancer)0 | GSE203612 | Breast cancer | BRCA | DCIS,ductal carcinoma in situ, solid type, microinvasion, pseudoangiomatosis, proliferative fibrocystic changes and sclerosing adenosis with calcification | Primary tumor | GSE203612 | 2022/8/5 | https://doi.org/10.1038/s41588-022-01141-9 | Visium |
| GSM6177603_NYU_BRCA(Breast cancer)2 | GSE203612 | Breast cancer | BRCA | ILC,invasive lobular carcinoma, pleomorphic and solid types with focal necrosis, apocrine and signet ring features, lobular carcinoma in situ classic type | Primary tumor | GSE203612 | 2022/8/5 | https://doi.org/10.1038/s41588-022-01141-9 | Visium |
| HCC-1L | Pmid: 34919432 | Hepatocellular carcinoma | HCC | None | Primary tumor | Pmid: 34919432 | 2021/12/17 | https://doi.org/10.1126/sciadv.abg3750 | Visium |
| HCC-2L | Pmid: 34919432 | Hepatocellular carcinoma | HCC | None | Primary tumor | Pmid: 34919432 | 2021/12/17 | https://doi.org/10.1126/sciadv.abg3750 | Visium |
| HCC-3L | Pmid: 34919432 | Hepatocellular carcinoma | HCC | None | Primary tumor | Pmid: 34919432 | 2021/12/17 | https://doi.org/10.1126/sciadv.abg3750 | Visium |
| HCC-4L | Pmid: 34919432 | Hepatocellular carcinoma | HCC | None | Primary tumor | Pmid: 34919432 | 2021/12/17 | https://doi.org/10.1126/sciadv.abg3750 | Visium |
| HCC2 | Pmid: 35673582 | Hepatocellular carcinoma | HCC | None | Primary tumor | Pmid: 35673582 | 2022/5/16 | https://doi.org/10.7150/thno.71873 | Visium |
| HCC3 | Pmid: 35673582 | Hepatocellular carcinoma | HCC | None | Primary tumor | Pmid: 35673582 | 2022/5/16 | https://doi.org/10.7150/thno.71873 | Visium |
| HCC_P1 | Pmid: 36708811 | Hepatocellular carcinoma | HCC | None response to PD-1 treatment/PFS:2months/etiology:HBV/male/53 years old | Primary tumor | Pmid: 36708811 | 2023/1/26 | https://doi.org/10.1016/j.jhep.2023.01.011 | Visium |
| HCC_P3 | Pmid: 36708811 | Hepatocellular carcinoma | HCC | None response to PD-1 treatment/PFS:1months/etiology:HBV/male/46 years old | Primary tumor | Pmid: 36708811 | 2023/1/26 | https://doi.org/10.1016/j.jhep.2023.01.011 | Visium |
| HCC_P7 | Pmid: 36708811 | Hepatocellular carcinoma | HCC | Response to PD-1 treatment/PFS:7months/etiology:HBV/male/68 years old | Primary tumor | Pmid: 36708811 | 2023/1/26 | https://doi.org/10.1016/j.jhep.2023.01.011 | Visium |
| HCC_P8 | Pmid: 36708811 | Hepatocellular carcinoma | HCC | None response to PD-1 treatment/PFS:1months/etiology:HBV/male/65 years old | Primary tumor | Pmid: 36708811 | 2023/1/26 | https://doi.org/10.1016/j.jhep.2023.01.011 | Visium |
| HCC_P9 | Pmid: 36708811 | Hepatocellular carcinoma | HCC | Response to PD-1 treatment/PFS:7months/etiology:HBV/male/41 years old | Primary tumor | Pmid: 36708811 | 2023/1/26 | https://doi.org/10.1016/j.jhep.2023.01.011 | Visium |
| HCC_P10 | Pmid: 36708811 | Hepatocellular carcinoma | HCC | Response to PD-1 treatment/PFS:12months/etiology:HBV/male/38 years old | Primary tumor | Pmid: 36708811 | 2023/1/26 | https://doi.org/10.1016/j.jhep.2023.01.011 | Visium |
| HCC_P15 | Pmid: 36708811 | Hepatocellular carcinoma | HCC | None response to PD-1 treatment/PFS:11months/etiology:HBV/male/52years old | Primary tumor | Pmid: 36708811 | 2023/1/26 | https://doi.org/10.1016/j.jhep.2023.01.011 | Visium |
| CRC1 | HRA000979 | Colorectal cancer | CRC | Male/66 years old/Stage II |  | HRA000979 | 2022/2/9 | https://doi.org/10.1038/s41467-022-29366-6 | Visium |
| CRC2 | HRA000979 | Colorectal cancer | CRC | Female/68 years old/Stage III |  | HRA000979 | 2022/2/9 | https://doi.org/10.1038/s41467-022-29366-6 | Visium |
| M1015 | in-house | Colorectal cancer | CRC | Female/71 years old/Stage II |  |  |  |  | Visium |
| M1016 | in-house | Colorectal cancer | CRC | Male/56 years old/Stage II |  |  |  |  | Visium |
| M1018 | in-house | Colorectal cancer | CRC | Male/66 years old/Stage II |  |  |  |  | Visium |
| M1022 | in-house | Colorectal cancer | CRC | Female/54 years old/Stage IV |  |  |  |  | Visium |
| M1026 | in-house | Colorectal cancer | CRC | Male/48 years old/Stage II |  |  |  |  | Visium |
| M1037 | in-house | Colorectal cancer | CRC | Male/32 years old/Stage IV |  |  |  |  | Visium |
| M1042 | in-house | Colorectal cancer | CRC | Male/58 years old/Stage II |  |  |  |  | Visium |
| M1051 | in-house | Colorectal cancer | CRC | Female/51 years old/Stage II |  |  |  |  | Visium |
| M1054 | in-house | Colorectal cancer | CRC | Female/63 years old/Stage II |  |  |  |  | Visium |
| M1055 | in-house | Colorectal cancer | CRC | Male/45 years old/Stage III |  |  |  |  | Visium |
| M1058 | in-house | Colorectal cancer | CRC | Male/72 years old/Stage II |  |  |  |  | Visium |
| M1066 | in-house | Colorectal cancer | CRC | Male/71 years old/Stage IV |  |  |  |  | Visium |
| M1067 | in-house | Colorectal cancer | CRC | Male/68 years old/Stage III |  |  |  |  | Visium |
| M1074 | in-house | Colorectal cancer | CRC | Female/57 years old/Stage IV |  |  |  |  | Visium |
| M1083 | in-house | Colorectal cancer | CRC | Female/71 years old/Stage II |  |  |  |  | Visium |
| M1084 | in-house | Colorectal cancer | CRC | Female/60 years old/Stage III |  |  |  |  | Visium |
| M1088 | in-house | Colorectal cancer | CRC | Female/54 years old/Stage IV |  |  |  |  | Visium |
| M1111 | in-house | Colorectal cancer | CRC | Male/67 years old/Stage II |  |  |  |  | Visium |
| M1112 | in-house | Colorectal cancer | CRC | Male/53 years old/Stage II |  |  |  |  | Visium |
| M1115 | in-house | Colorectal cancer | CRC | Female/64 years old/Stage II |  |  |  |  | Visium |
| M1124 | in-house | Colorectal cancer | CRC | Female/74 years old/Stage III |  |  |  |  | Visium |
| M1133 | in-house | Colorectal cancer | CRC | Male/60 years old/Stage II |  |  |  |  | Visium |
| M2006 | in-house | Colorectal cancer | CRC | Female/69 years old/Stage III |  |  |  |  | Visium |
| A2 | zenodo.5511762 | HER2+ breast cancer | HER2 |  |  | zenodo.5511762 | 2021/10/14 | https://doi.org/10.1038/s41467-021-26271-2 | ST |
| A3 | zenodo.5511762 | HER2+ breast cancer | HER2 |  |  | zenodo.5511762 | 2021/10/14 | https://doi.org/10.1038/s41467-021-26271-2 | ST |
| A4 | zenodo.5511762 | HER2+ breast cancer | HER2 |  |  | zenodo.5511762 | 2021/10/14 | https://doi.org/10.1038/s41467-021-26271-2 | ST |
| A5 | zenodo.5511762 | HER2+ breast cancer | HER2 |  |  | zenodo.5511762 | 2021/10/14 | https://doi.org/10.1038/s41467-021-26271-2 | ST |
| A6 | zenodo.5511762 | HER2+ breast cancer | HER2 |  |  | zenodo.5511762 | 2021/10/14 | https://doi.org/10.1038/s41467-021-26271-2 | ST |
| B1 | zenodo.5511762 | HER2+ breast cancer | HER2 |  |  | zenodo.5511762 | 2021/10/14 | https://doi.org/10.1038/s41467-021-26271-2 | ST |
| B2 | zenodo.5511762 | HER2+ breast cancer | HER2 |  |  | zenodo.5511762 | 2021/10/14 | https://doi.org/10.1038/s41467-021-26271-2 | ST |
| B3 | zenodo.5511762 | HER2+ breast cancer | HER2 |  |  | zenodo.5511762 | 2021/10/14 | https://doi.org/10.1038/s41467-021-26271-2 | ST |
| B4 | zenodo.5511762 | HER2+ breast cancer | HER2 |  |  | zenodo.5511762 | 2021/10/14 | https://doi.org/10.1038/s41467-021-26271-2 | ST |
| B5 | zenodo.5511762 | HER2+ breast cancer | HER2 |  |  | zenodo.5511762 | 2021/10/14 | https://doi.org/10.1038/s41467-021-26271-2 | ST |
| B6 | zenodo.5511762 | HER2+ breast cancer | HER2 |  |  | zenodo.5511762 | 2021/10/14 | https://doi.org/10.1038/s41467-021-26271-2 | ST |
| C1 | zenodo.5511762 | HER2+ breast cancer | HER2 |  |  | zenodo.5511762 | 2021/10/14 | https://doi.org/10.1038/s41467-021-26271-2 | ST |
| C2 | zenodo.5511762 | HER2+ breast cancer | HER2 |  |  | zenodo.5511762 | 2021/10/14 | https://doi.org/10.1038/s41467-021-26271-2 | ST |
| C3 | zenodo.5511762 | HER2+ breast cancer | HER2 |  |  | zenodo.5511762 | 2021/10/14 | https://doi.org/10.1038/s41467-021-26271-2 | ST |
| C4 | zenodo.5511762 | HER2+ breast cancer | HER2 |  |  | zenodo.5511762 | 2021/10/14 | https://doi.org/10.1038/s41467-021-26271-2 | ST |
| C5 | zenodo.5511762 | HER2+ breast cancer | HER2 |  |  | zenodo.5511762 | 2021/10/14 | https://doi.org/10.1038/s41467-021-26271-2 | ST |
| C6 | zenodo.5511762 | HER2+ breast cancer | HER2 |  |  | zenodo.5511762 | 2021/10/14 | https://doi.org/10.1038/s41467-021-26271-2 | ST |
| D1 | zenodo.5511762 | HER2+ breast cancer | HER2 |  |  | zenodo.5511762 | 2021/10/14 | https://doi.org/10.1038/s41467-021-26271-2 | ST |
| D2 | zenodo.5511762 | HER2+ breast cancer | HER2 |  |  | zenodo.5511762 | 2021/10/14 | https://doi.org/10.1038/s41467-021-26271-2 | ST |
| D3 | zenodo.5511762 | HER2+ breast cancer | HER2 |  |  | zenodo.5511762 | 2021/10/14 | https://doi.org/10.1038/s41467-021-26271-2 | ST |
| D4 | zenodo.5511762 | HER2+ breast cancer | HER2 |  |  | zenodo.5511762 | 2021/10/14 | https://doi.org/10.1038/s41467-021-26271-2 | ST |
| D5 | zenodo.5511762 | HER2+ breast cancer | HER2 |  |  | zenodo.5511762 | 2021/10/14 | https://doi.org/10.1038/s41467-021-26271-2 | ST |
| D6 | zenodo.5511762 | HER2+ breast cancer | HER2 |  |  | zenodo.5511762 | 2021/10/14 | https://doi.org/10.1038/s41467-021-26271-2 | ST |
| E1 | zenodo.5511762 | HER2+ breast cancer | HER2 |  |  | zenodo.5511762 | 2021/10/14 | https://doi.org/10.1038/s41467-021-26271-2 | ST |
| E2 | zenodo.5511762 | HER2+ breast cancer | HER2 |  |  | zenodo.5511762 | 2021/10/14 | https://doi.org/10.1038/s41467-021-26271-2 | ST |
| E3 | zenodo.5511762 | HER2+ breast cancer | HER2 |  |  | zenodo.5511762 | 2021/10/14 | https://doi.org/10.1038/s41467-021-26271-2 | ST |
| F1 | zenodo.5511762 | HER2+ breast cancer | HER2 |  |  | zenodo.5511762 | 2021/10/14 | https://doi.org/10.1038/s41467-021-26271-2 | ST |
| F2 | zenodo.5511762 | HER2+ breast cancer | HER2 |  |  | zenodo.5511762 | 2021/10/14 | https://doi.org/10.1038/s41467-021-26271-2 | ST |
| F3 | zenodo.5511762 | HER2+ breast cancer | HER2 |  |  | zenodo.5511762 | 2021/10/14 | https://doi.org/10.1038/s41467-021-26271-2 | ST |
| G1 | zenodo.5511762 | HER2+ breast cancer | HER2 |  |  | zenodo.5511762 | 2021/10/14 | https://doi.org/10.1038/s41467-021-26271-2 | ST |
| G2 | zenodo.5511762 | HER2+ breast cancer | HER2 |  |  | zenodo.5511762 | 2021/10/14 | https://doi.org/10.1038/s41467-021-26271-2 | ST |
| G3 | zenodo.5511762 | HER2+ breast cancer | HER2 |  |  | zenodo.5511762 | 2021/10/14 | https://doi.org/10.1038/s41467-021-26271-2 | ST |
| P2_ST_rep1 | GSE144240 | Cutaneous squamous cell carcinoma | cSCC |  |  | GSE144240 | 2020/7/23 | https://doi.org/10.1016/j.cell.2020.05.039 | ST |
| P2_ST_rep2 | GSE144240 | Cutaneous squamous cell carcinoma | cSCC |  |  | GSE144240 | 2020/7/23 | https://doi.org/10.1016/j.cell.2020.05.039 | ST |
| P2_ST_rep3 | GSE144240 | Cutaneous squamous cell carcinoma | cSCC |  |  | GSE144240 | 2020/7/23 | https://doi.org/10.1016/j.cell.2020.05.039 | ST |
| P5_ST_rep1 | GSE144240 | Cutaneous squamous cell carcinoma | cSCC |  |  | GSE144240 | 2020/7/23 | https://doi.org/10.1016/j.cell.2020.05.039 | ST |
| P5_ST_rep2 | GSE144240 | Cutaneous squamous cell carcinoma | cSCC |  |  | GSE144240 | 2020/7/23 | https://doi.org/10.1016/j.cell.2020.05.039 | ST |
| P5_ST_rep3 | GSE144240 | Cutaneous squamous cell carcinoma | cSCC |  |  | GSE144240 | 2020/7/23 | https://doi.org/10.1016/j.cell.2020.05.039 | ST |
| P9_ST_rep1 | GSE144240 | Cutaneous squamous cell carcinoma | cSCC |  |  | GSE144240 | 2020/7/23 | https://doi.org/10.1016/j.cell.2020.05.039 | ST |
| P9_ST_rep2 | GSE144240 | Cutaneous squamous cell carcinoma | cSCC |  |  | GSE144240 | 2020/7/23 | https://doi.org/10.1016/j.cell.2020.05.039 | ST |
| P9_ST_rep3 | GSE144240 | Cutaneous squamous cell carcinoma | cSCC |  |  | GSE144240 | 2020/7/23 | https://doi.org/10.1016/j.cell.2020.05.039 | ST |
| P10_ST_rep1 | GSE144240 | Cutaneous squamous cell carcinoma | cSCC |  |  | GSE144240 | 2020/7/23 | https://doi.org/10.1016/j.cell.2020.05.039 | ST |
| P10_ST_rep2 | GSE144240 | Cutaneous squamous cell carcinoma | cSCC |  |  | GSE144240 | 2020/7/23 | https://doi.org/10.1016/j.cell.2020.05.039 | ST |
| P10_ST_rep3 | GSE144240 | Cutaneous squamous cell carcinoma | cSCC |  |  | GSE144240 | 2020/7/23 | https://doi.org/10.1016/j.cell.2020.05.039 | ST |

**Table S2.** Pearson correlation coefficient (Rp) between the spatial gene expression profiles (GEPs) from ground truth of ST data and predicted by different models.

| **Spatial Variable Genes** | | | | | |
| --- | --- | --- | --- | --- | --- |
| Cancer type | Model name | Sample number | Target gene number | Mean correlation | 95% CI |
| BRCA | HiST | 42 | 470 | 0.83 | 0.79–0.87 |
|  | EGNv2 | 42 | 470 | 0.41 | 0.36–0.46 |
|  | IGI-DL | 42 | 470 | 0.29 | 0.24–0.34 |
|  | HisToGene | 42 | 470 | 0.13 | 0.11–0.15 |
|  | ThItoGene | 42 | 470 | 0.11 | 0.08–0.14 |
|  | ST-Net | 42 | 470 | 0.00 | -0.07–0.07 |
| CRC | HiST | 25 | 346 | 0.62 | 0.58–0.66 |
|  | EGNv2 | 25 | 346 | 0.34 | 0.29–0.39 |
|  | IGI-DL | 25 | 346 | 0.33 | 0.27–0.39 |
|  | HisToGene | 25 | 346 | 0.07 | 0.06–0.08 |
|  | ThItoGene | 25 | 346 | 0.04 | 0.02–0.06 |
|  | ST-Net | 25 | 346 | 0.04 | -0.01–0.09 |
| HCC | HiST | 13 | 448 | 0.63 | 0.57–0.69 |
|  | EGNv2 | 13 | 448 | 0.29 | 0.21–0.37 |
|  | IGI-DL | 13 | 448 | 0.23 | 0.15–0.31 |
|  | HisToGene | 13 | 448 | 0.09 | 0.05–0.13 |
|  | ThItoGene | 13 | 448 | 0.02 | 0.00–0.04 |
|  | ST-Net | 13 | 448 | 0.04 | -0.02–0.10 |
| KIRC | HiST | 14 | 181 | 0.68 | 0.59–0.77 |
|  | EGNv2 | 14 | 181 | 0.19 | 0.12–0.26 |
|  | IGI-DL | 14 | 181 | 0.11 | 0.03–0.19 |
|  | HisToGene | 14 | 181 | 0.00 | 0.00–0.00 |
|  | ThItoGene | 14 | 181 | 0.01 | -0.02–0.04 |
|  | ST-Net | 14 | 181 | 0.03 | -0.03–0.09 |
| OV | HiST | 8 | 144 | 0.79 | 0.72–0.86 |
|  | EGNv2 | 8 | 144 | 0.22 | 0.11–0.33 |
|  | IGI-DL | 8 | 144 | 0.15 | 0.05–0.25 |
|  | HisToGene | 8 | 144 | 0.02 | 0.01–0.03 |
|  | ThItoGene | 8 | 144 | 0.03 | -0.01–0.07 |
|  | ST-Net | 8 | 144 | 0.10 | -0.03–0.23 |
| HER2 | HiST | 32 | 785 | 0.45 | 0.41–0.49 |
|  | EGNv2 | 32 | 785 | 0.17 | 0.12–0.22 |
|  | Hist2ST | 32 | 785 | 0.16 | 0.13–0.19 |
|  | HisToGene | 32 | 785 | 0.08 | 0.06–0.10 |
|  | THItoGene | 32 | 785 | 0.19 | 0.16–0.22 |
|  | ST-Net | 32 | 785 | 0.00 | -0.03–0.03 |
| cSCC | HiST | 12 | 171 | 0.41 | 0.33–0.49 |
|  | EGNv2 | 12 | 171 | 0.24 | 0.17–0.31 |
|  | Hist2ST | 12 | 171 | 0.17 | 0.13–0.21 |
|  | HisToGene | 12 | 171 | 0.08 | 0.05–0.11 |
|  | THItoGene | 12 | 171 | 0.24 | 0.20–0.28 |
|  | ST-Net | 12 | 171 | 0.06 | -0.01–0.13 |
| **IGI-DL Selected Genes** | | | | | |
| Cancer type | Model name | Sample number | Target gene number | Mean correlation | 95% CI |
| HER2 | HiST | 32 | 187 | 0.72 | 0.69–0.75 |
|  | IGI-DL | 32 | 187 | 0.23 | NA |
| cSCC | HiST | 12 | 487 | 0.60 | 0.52–0.68 |
|  | IGI-DL | 12 | 487 | 0.20 | NA |

**Table S3.** The clinical characteristics of the five cancer types obtained from TCGA cohorts were used for survival analysis.

| **TCGA-BRCA** | | |
| --- | --- | --- |
| Characteristics |  | Summary(N=1052) |
| Age at index |  | 58.5±13.2 years |
| Gender | Male Female | 12(1.1%) 1040(98.9%) |
| Status | Dead Alive | 144(15.9%)  908(84.1%) |
| Stage | Stage I  Stage II  Stage III  Stage IV Stage X | 175(16.6%) 606(57.6%) 239(22.7%) 19(1.8%) 13(1.3%) |
| **TCGA-CRC** | | |
| Characteristics |  | Summary(N=434) |
| Age at index |  | 67.0±13.0 years |
| Gender | Male Female | 224(51.6%) 210(48.4%) |
| Status | Dead Alive | 92(21.2%)  342(78.8%) |
| Stage | Stage I  Stage II  Stage III  Stage IV | 73(16.8%) 172(39.6%) 125(28.8%) 64(14.8%) |
| **TCGA-HCC** | | |
| Characteristics |  | Summary(N=341) |
| Age at index |  | 59.1±13.5 years |
| Gender | Male Female | 232(68.0%) 109(32.0%) |
| Status | Dead Alive | 115(33.7%)  226(66.3%) |
| Stage | Stage I  Stage II  Stage III  Stage IV | 170(49.9%) 83(24.3%) 83(24.3%) 5(1.5%) |
| **TCGA-KIRC** | | |
| Characteristics |  | Summary(N=512) |
| Age at index |  | 60.6±12.1 years |
| Gender | Male Female | 330(64.5%) 182(35.5%) |
| Status | Dead Alive | 170(33.2%)  342(66.8%) |
| Stage | Stage I  Stage II  Stage III  Stage IV | 257(50.2%) 57(11.1%) 119(23.3%) 79(15.4%) |
| **TCGA-OV** | | |
| Characteristics |  | Summary(N=104) |
| Age at index |  | 60.9±11.2 years |
| Status | Dead Alive | 72(33.2%)  32(66.8%) |
| Stage | Stage I  Stage II  Stage III  Stage IV | 2(1.9%) 4(3.8%) 74(71.2%) 24(23.1%) |

**Table S4.** Clinical information on patients receiving immunotherapy for liver cancer.

| **Patient_id** | **Age** | **Gender** | **Start date** | **End date** | **PFS(day)** | **Response** |
| --- | --- | --- | --- | --- | --- | --- |
| patient1 | 70 | male | NA | NA | NA | NR |
| patient2-2 | 42 | female | NA | NA | NA | NR |
| patient2-1 | 42 | female | NA | NA | NA | NR |
| patient3 | 58 | female | NA | NA | NA | NR |
| patient4 | 56 | male | 2021/6/9 | 2023/2/16 | 617 | NR |
| patient5 | 69 | male | 2023/5/23 | 2023/7/10 | 48 | NR |
| patient6 | 55 | male | 2023/9/29 | 2024/1/8 | 101 | NR |
| patient7 | 27 | male | NA | NA | NA | NR |
| patient8 | NA | NA | 2024/2/2 | 2024/3/18 | 45 | NR |
| patient9 | 55 | male | 2023/11/13 | 2023/12/15 | 32 | NR |
| patient10 | 60 | male | 2020/10/21 | 2020/11/26 | 36 | NR |
| patient11 | 73 | male | 2020/10/19 | 2021/7/21 | 275 | NR |
| patient12 | 70 | male | 2022/3/13 | 2023/2/2 | 326 | NR |
| patient13 | 32 | male | 2020/8/21 | 2021/12/24 | 490 | R |
| patient14 | 62 | male | 2020/5/19 | 2020/8/24 | 97 | NR |
| patient15 | 57 | male | 2020/9/19 | 2021/2/19 | 153 | NR |
| patient16 | 78 | male | NA | NA | NA | NR |
| patient17 | 48 | male | NA | NA | NA | NR |
| patient18 | 75 | female | 2020/7/24 | 2021/9/24 | 427 | NR |
| patient19 | 59 | male | 2021/8/11 | 2023/1/6 | 513 | NR |
| patient20 | 70 | male | 2020/7/20 | 2021/8/31 | 407 | NR |
| patient21 | 68 | male | NA | NA | NA | NR |
| patient22 | 70 | male | 2021/5/25 | 2022/6/9 | 380 | NR |
| patient23-2 | 55 | male | 2024/2/2 | 2024/9/19 | 230 | R |
| patient23-1 | 55 | male | 2024/2/2 | 2024/9/19 | 230 | R |
| patient24 | 65 | male | 2021/3/11 | 2021/11/18 | 252 | NR |
| patient25 | 51 | female | 2021/3/9 | 2024/8/26 | 1266 | R |
| patient26 | 49 | male | 2021/4/26 | 2022/7/22 | 452 | NR |
| patient27 | 60 | male | 2020/10/21 | 2021/3/10 | 140 | NR |
| patient28 | 54 | male | 2021/5/12 | 2021/8/3 | 83 | NR |
| patient29 | 64 | male | 2021/5/28 | 2021/11/9 | 165 | NR |
| patient30 | 55 | male | 2021/6/2 | 2022/3/1 | 272 | R |
| patient31 | 61 | male | 2021/7/12 | 2021/9/29 | 79 | NR |
| patient32 | 66 | female | 2022/5/16 | 2023/1/28 | 257 | NR |
| patient33 | 50 | male | 2021/10/21 | 2022/6/1 | 223 | NR |
| patient34 | 57 | female | 2022/4/14 | 2022/9/7 | 146 | NR |
| patient35 | 58 | male | 2021/7/9 | 2022/6/8 | 334 | NR |
| patient36 | 66 | female | 2022/2/13 | 2022/8/5 | 173 | NR |
| patient37 | 54 | male | 2021/8/3 | 2022/8/5 | 367 | NR |
| patient38 | 50 | male | 2021/9/11 | 2024/9/11 | 1096 | R |
| patient39 | 52 | female | 2021/8/26 | 2022/7/25 | 333 | NR |
| patient40 | 52 | male | 2022/3/25 | 2022/8/18 | 146 | NR |
| patient41 | 73 | male | 2022/4/13 | 2022/10/24 | 194 | NR |
| patient42 | 26 | male | 2022/2/8 | 2022/5/6 | 87 | NR |
| patient43 | 66 | female | 2022/2/16 | 2022/5/10 | 83 | NR |
| patient44 | 59 | male | 2021/10/29 | 2022/12/21 | 418 | NR |
| patient45 | 58 | male | 2021/12/15 | 2022/3/5 | 80 | NR |
| patient46-2 | 74 | male | 2021/10/24 | 2022/8/30 | 310 | NR |
| patient46-1 | 74 | male | 2021/10/24 | 2022/8/30 | 310 | NR |
| patient47 | 55 | male | 2022/1/28 | 2022/5/11 | 103 | NR |
| patient48 | 72 | male | NA | NA | NA | NR |
| patient49 | 61 | male | 2022/9/1 | 2023/5/29 | 270 | NR |
| patient50 | 32 | male | 2022/5/9 | 2022/7/27 | 79 | NR |
| patient51 | 60 | male | 2022/1/17 | 2024/5/22 | 856 | NR |
| patient52 | 52 | male | 2022/5/13 | 2022/7/12 | 60 | NR |
| patient53 | 55 | male | 2022/2/18 | 2022/8/4 | 167 | NR |
| patient54 | 75 | male | 2024/5/6 | 2024/7/17 | 72 | NR |
| patient55 | 72 | male | 2022/5/6 | 2022/6/16 | 41 | NR |
| patient56 | 68 | male | 2023/6/5 | 2024/5/8 | 338 | NR |
| patient57 | 71 | male | 2023/3/23 | 2023/10/26 | 217 | NR |
| patient58 | 61 | male | 2022/7/23 | 2023/12/8 | 503 | NR |
| patient59 | 58 | male | NA | NA | NA | NR |
| patient60 | 71 | male | 2022/12/15 | 2023/9/18 | 277 | NR |
| patient61 | 75 | male | 2023/10/27 | 2024/2/1 | 97 | NR |
| patient62 | 55 | male | 2023/6/1 | 2024/5/8 | 342 | NR |
| patient63 | 58 | male | 2022/12/18 | 2023/2/7 | 51 | NR |
| patient64 | 54 | male | 2023/5/15 | 2024/9/25 | 499 | R |
| patient65 | 54 | male | 2022/12/17 | 2023/5/15 | 149 | NR |
| patient66-2 | 55 | male | 2024/2/22 | 2024/8/26 | 186 | NR |
| patient66-1 | 55 | male | 2024/2/22 | 2024/8/26 | 186 | NR |
| patient67 | 61 | male | 2023/6/30 | 2023/9/25 | 87 | NR |
| patient68 | 60 | male | 2023/7/21 | 2023/11/23 | 125 | NR |
| patient69 | 47 | male | 2024/1/17 | 2024/9/18 | 245 | R |
| patient70 | 55 | male | 2023/8/29 | 2024/5/8 | 253 | NR |
| patient71 | 70 | female | 2023/11/30 | 2024/1/11 | 42 | NR |
| patient72 | 61 | male | 2024/1/30 | 2024/4/23 | 84 | NR |
| patient73 | NA | NA | 2024/2/2 | 2024/3/18 | 45 | NR |
| patient74 | 53 | female | 2024/2/28 | 2024/3/8 | 9 | NR |
| patient75-2 | 37 | male | 2023/12/8 | 2024/3/9 | 92 | NR |
| patient75-1 | 37 | male | 2023/12/8 | 2024/3/9 | 92 | NR |
| patient76-2 | NA | NA | NA | NA | NA | NR |
| patient76-1 | NA | NA | NA | NA | NA | NR |
| patient77 | 52 | male | 2024/2/5 | 2024/9/3 | 211 | R |
| patient78 | 71 | male | 2024/2/29 | 2024/8/20 | 173 | R |
| patient79 | 53 | male | 2021/9/6 | 2024/1/9 | 855 | R |
| patient80 | 60 | female | 2020/10/23 | 2024/8/27 | 1404 | R |
| patient81 | 68 | male | 2023/7/18 | 2024/3/8 | 234 | R |
| patient82 | 61 | male | 2022/11/30 | 2024/9/21 | 661 | R |
| patient83 | 36 | male | 2022/3/31 | 2022/8/9 | 131 | NR |
| patient84 | 72 | male | 2023/6/21 | 2024/9/18 | 455 | R |
| patient85 | 41 | male | 2021/10/27 | 2022/7/4 | 250 | R |
| patient86 | 76 | female | 2023/3/1 | 2024/9/18 | 567 | R |
| patient87 | 49 | male | 2023/2/21 | 2024/9/18 | 575 | R |
| patient88 | 39 | male | 2023/6/12 | 2024/8/28 | 443 | R |
| patient89 | 49 | male | 2023/5/19 | 2024/1/30 | 256 | R |
| patient90 | 54 | male | 2024/1/24 | 2024/8/20 | 209 | R |
| patient91 | 75 | male | 2020/10/24 | 2021/3/17 | 144 | NR |
| patient92 | 51 | male | 2020/7/23 | 2021/3/1 | 221 | NR |
| patient93 | NA | NA | NA | NA | NA | NR |
| patient94 | 52 | male | 2020/12/8 | 2022/1/19 | 407 | NR |
| patient95 | 78 | female | 2020/8/25 | 2022/10/2 | 768 | R |
| patient96 | 37 | male | NA | NA | NA | NR |
| patient97 | 76 | male | 2020/5/11 | 2020/8/7 | 88 | NR |
| patient98 | 76 | male | 2022/12/10 | 2024/7/22 | 590 | NR |
| patient99-2 | 57 | male | NA | NA | NA | NR |
| patient99-1 | 57 | male | NA | NA | NA | NR |
| patient100-2 | 53 | male | 2021/1/12 | 2022/6/29 | 533 | NR |
| patient100-3 | 53 | male | 2021/1/12 | 2022/6/29 | 533 | NR |
| patient100-4 | 53 | male | 2021/1/12 | 2022/6/29 | 533 | NR |
| patient100-1 | 53 | male | 2021/1/12 | 2022/6/29 | 533 | NR |
| patient101 | 36 | male | 2021/11/19 | 2024/3/29 | 861 | R |
| patient102 | 72 | male | 2021/7/19 | 2021/11/12 | 116 | NR |
| patient103 | 50 | male | 2021/6/7 | 2023/12/13 | 919 | R |
| patient104 | 59 | male | 2022/4/29 | 2023/7/25 | 452 | NR |
| patient105 | 59 | male | 2021/7/17 | 2021/8/13 | 27 | NR |
| patient106 | 33 | male | 2023/5/16 | 2024/9/12 | 485 | NR |
| patient107 | 69 | male | 2022/4/19 | 2022/5/23 | 34 | NR |
| patient108 | 55 | male | 2022/9/25 | 2024/8/8 | 683 | R |
| patient109 | 59 | male | 2022/10/24 | 2023/4/7 | 165 | NR |
| patient110 | 71 | female | 2023/2/20 | 2024/6/27 | 493 | NR |
| patient111 | 55 | male | 2022/8/18 | 2022/11/15 | 89 | R |
| patient112 | 55 | male | 2023/8/10 | 2024/4/26 | 260 | R |
| patient113 | 42 | male | 2024/4/18 | 2024/8/22 | 126 | NR |
| patient114 | 60 | male | 2022/12/18 | 2023/2/13 | 57 | NR |
| patient115 | 53 | male | 2022/10/14 | 2024/2/27 | 501 | NR |
| patient116 | 51 | male | 2022/11/2 | 2022/12/2 | 30 | NR |
| patient117 | 56 | male | 2023/1/6 | 2024/8/13 | 585 | R |
| patient118 | 54 | male | NA | NA | NA | NR |
| patient119 | 66 | male | 2023/5/23 | 2024/7/23 | 427 | R |
| patient120 | 62 | male | 2023/11/3 | 2023/12/13 | 40 | NR |
| patient121 | 61 | male | 2024/3/14 | 2024/6/7 | 85 | NR |

**Table S5.** Hyperparameters of training deep learning models for different task.

| **Tumor spot identification model hyperparameters** | | | | | |
| --- | --- | --- | --- | --- | --- |
| **Dataset** | **mask ch** | **img ch** | **epochs** | **lr** | **weight decay** |
| BRCA | 1 | 768 | 200 | 0.001 | 0.0001 |
| CRC | 1 | 768 | 200 | 0.001 | 0.0001 |
| HCC | 1 | 768 | 200 | 0.001 | 0.0001 |
| KIRC | 1 | 768 | 200 | 0.001 | 0.0001 |
| OV | 1 | 768 | 200 | 0.001 | 0.0001 |

| **Spatial GEP prediction model hyperparameters** | | | | |
| --- | --- | --- | --- | --- |
| **Dataset** | **img ch** | **epochs** | **lr** | **weight decay** |
| BRCA | 768 | 200 | 0.001 | 0.0001 |
| CRC | 768 | 200 | 0.001 | 0.0001 |
| HCC | 768 | 200 | 0.001 | 0.0001 |
| KIRC | 768 | 200 | 0.001 | 0.0001 |
| OV | 768 | 200 | 0.001 | 0.0001 |
| HER2 | 768 | 200 | 0.001 | 0.0001 |
| cSCC | 768 | 200 | 0.001 | 0.0001 |

| **Survival model hyperparameters** | | | | | | | | | | | |
| --- | --- | --- | --- | --- | --- | --- | --- | --- | --- | --- | --- |
| **Dataset** | **Model** | **torch seed** | **kfold seed** | **drop path rate** | **depths** | **dims** | **clinical col** | **loss** | **epochs** | **lr** | **HE dim** |
|  | spatial gene expression | 140 | 92 | 0.4 | [2, 2, 2, 2] | [2, 3, 4, 5] | 7 | coxph | 300 | 0.012 | 768 |
| BRCA | HE feature | 140 | 92 | 0.4 | [2, 2, 2, 2] | [2, 3, 4, 5] | 7 | coxph | 300 | 0.012 | 768 |
|  | Resnet50 | 140 | 92 | 0.4 | [2, 2, 2, 2] | [2, 3, 4, 5] | 7 | coxph | 300 | 0.012 | 2048 |
|  | spatial gene expression | 1 | 15 | 0.4 | [2, 2, 2, 2] | [3, 4, 5, 6] | 6 | coxph | 200 | 0.015 | 768 |
| CRC | HE feature | 1 | 15 | 0.4 | [2, 2, 2, 2] | [3, 4, 5, 6] | 6 | coxph | 200 | 0.015 | 768 |
|  | Resnet50 | 1 | 15 | 0.4 | [2, 2, 2, 2] | [3, 4, 5, 6] | 6 | coxph | 200 | 0.015 | 2048 |
|  | spatial gene expression | 648 | 32 | 0.4 | [2, 2, 2, 2] | [1, 2, 3, 4] | 7 | coxph | 150 | 0.02 | 768 |
| HCC | HE feature | 648 | 32 | 0.4 | [2, 2, 2, 2] | [1, 2, 3, 4] | 7 | coxph | 150 | 0.02 | 768 |
|  | Resnet50 | 648 | 32 | 0.4 | [2, 2, 2, 2] | [1, 2, 3, 4] | 7 | coxph | 150 | 0.02 | 2048 |
|  | spatial gene expression | 123 | 8 | 0.4 | [2, 2, 2, 2] | [2, 3, 4, 5] | 6 | coxph | 300 | 0.012 | 768 |
| KIRC | HE feature | 123 | 8 | 0.4 | [2, 2, 2, 2] | [2, 3, 4, 5] | 6 | coxph | 300 | 0.012 | 768 |
|  | Resnet50 | 123 | 8 | 0.4 | [2, 2, 2, 2] | [2, 3, 4, 5] | 6 | coxph | 300 | 0.012 | 2048 |
|  | spatial gene expression | 159 | 25 | 0.4 | [2, 2, 2, 2] | [2, 3, 4, 5] | 5 | coxph | 300 | 0.012 | 768 |
| OV | HE feature | 159 | 25 | 0.4 | [2, 2, 2, 2] | [2, 3, 4, 5] | 5 | coxph | 300 | 0.012 | 768 |
|  | Resnet50 | 159 | 25 | 0.4 | [2, 2, 2, 2] | [2, 3, 4, 5] | 5 | coxph | 300 | 0.012 | 2048 |

| **Immune therapy outcome prediction model hyperparameters** | | | | | | |
| --- | --- | --- | --- | --- | --- | --- |
| **torch seed** | **kfold seed** | **drop path rate** | **depths** | **dims** | **epochs** | **lr** |
| 22 | 123 | 0.4 | [2, 2, 8, 2] | [16, 24, 32, 40] | 200 | 5.00E-05 |
